# Risk prediction models for incident type 2 diabetes in Chinese people with intermediate hyperglycemia: a systematic literature review and external validation study

**DOI:** 10.1186/s12933-022-01622-5

**Published:** 2022-09-13

**Authors:** Shishi Xu, Ruth L. Coleman, Qin Wan, Yeqing Gu, Ge Meng, Kun Song, Zumin Shi, Qian Xie, Jaakko Tuomilehto, Rury R. Holman, Kaijun Niu, Nanwei Tong

**Affiliations:** 1 Division of Endocrinology and Metabolism, Center for Diabetes and Metabolism Research, Laboratory of Diabetes and Islet Transplantation Research, West China Medical School, West China Hospital, Sichuan University, Guo Xue Lane 37, Chengdu, China; 2 Diabetes Trials Unit, Radcliffe Department of Medicine, University of Oxford, Oxford, UK; 3 Department of Endocrine and Metabolic Diseases, The Affiliated Hospital of Southwest Medical University, Luzhou, China; 4 Nutrition and Radiation Epidemiology Research Center, Institute of Radiation Medicine, Chinese Academy of Medical Sciences & Peking Union Medical College, Tianjin, China; 5 Nutritional Epidemiology Institute and School of Public Health, Tianjin Medical University, Tianjin, China; 6 Health Management Centre, Tianjin Medical University General Hospital, Tianjin, China; 7 Human Nutrition Department, College of Health Sciences, QU Health, Qatar University, Doha, Qatar; 8 Department of General Practice, People’s Hospital of LeShan, LeShan, China; 9 Department of Public Health, University of Helsinki, Helsinki, Finland; 10 Population Health Unit, Finnish Institute for Health and Welfare, Helsinki, Finland; 11 Saudi Diabetes Research Group, King Abdulaziz University, Jeddah, Saudi Arabia

**Keywords:** Risk prediction model, Type 2 diabetes, Intermediate hyperglycemia, Risk stratification, Primary prevention, Chinese population

## Abstract

**Background:**

People with intermediate hyperglycemia (IH), including impaired fasting glucose and/or impaired glucose tolerance, are at higher risk of developing type 2 diabetes (T2D) than those with normoglycemia. We aimed to evaluate the performance of published T2D risk prediction models in Chinese people with IH to inform them about the choice of primary diabetes prevention measures.

**Methods:**

A systematic literature search was conducted to identify Asian-derived T2D risk prediction models, which were eligible if they were built on a prospective cohort of Asian adults without diabetes at baseline and utilized routinely-available variables to predict future risk of T2D. These Asian-derived and five prespecified non-Asian derived T2D risk prediction models were divided into BASIC (clinical variables only) and EXTENDED (plus laboratory variables) versions, with validation performed on them in three prospective Chinese IH cohorts: ACE (n = 3241), Luzhou (n = 1333), and TCLSIH (n = 1702). Model performance was assessed in terms of discrimination (C-statistic) and calibration (Hosmer–Lemeshow test).

**Results:**

Forty-four Asian and five non-Asian studies comprising 21 BASIC and 46 EXTENDED T2D risk prediction models for validation were identified. The majority were at high (n = 43, 87.8%) or unclear (n = 3, 6.1%) risk of bias, while only three studies (6.1%) were scored at low risk of bias. BASIC models showed poor-to-moderate discrimination with C-statistics 0.52–0.60, 0.50–0.59, and 0.50–0.64 in the ACE, Luzhou, and TCLSIH cohorts respectively. EXTENDED models showed poor-to-acceptable discrimination with C-statistics 0.54–0.73, 0.52–0.67, and 0.59–0.78 respectively. Fifteen BASIC and 40 EXTENDED models showed poor calibration (*P* < 0.05), overpredicting or underestimating the observed diabetes risk. Most recalibrated models showed improved calibration but modestly-to-severely overestimated diabetes risk in the three cohorts. The *NAVIGATOR* model showed the best discrimination in the three cohorts but had poor calibration (*P* < 0.05).

**Conclusions:**

In Chinese people with IH, previously published BASIC models to predict T2D did not exhibit good discrimination or calibration. Several EXTENDED models performed better, but a robust Chinese T2D risk prediction tool in people with IH remains a major unmet need.

**Supplementary Information:**

The online version contains supplementary material available at 10.1186/s12933-022-01622-5.

## Background

People with intermediate hyperglycemia (IH), including impaired fasting glucose (IFG) and/or impaired glucose tolerance (IGT), are at higher risk of developing type 2 diabetes (T2D) than those with normoglycemia [1, 2]. However, this population comprises a heterogeneous group with differing diabetes incidence rates [2, 3]. Individualized risk estimation for T2D is important to help inform decision-making when considering measures for primary prevention of T2D.

Preventing diabetes is a particular challenge in China, which has the world’s largest population with IH [4]. Although several T2D risk prediction models exist, there is, unfortunately, no validated tool to predict the risk of T2D for Chinese people with IH. The risk stratification policy in China recommended currently to guide primary prevention measures in the IH population is based on a “simple” strategy (referred to as “Chinese IH risk stratification” below) rather than quantifying their absolute risk [5]. High-risk individuals are defined as those with combined IFG and IGT, or individuals with isolated IFG or isolated IGT but having at least one specified risk factor (i.e., overweight or obesity, family history of diabetes, gestational diabetes mellitus, dyslipidemia, hypertension, cardiovascular disease, non-alcoholic fatty liver disease, or polycystic ovarian syndrome), whereas those with isolated IFG or isolated IGT and without these specified risk factors are categorized as low risk.

We sought to identify an effective T2D risk estimation tool for people with IH by conducting an external validation study to evaluate the performance of existing risk prediction models using three independent prospective Chinese IH cohorts. Our primary focus was to examine Asian-derived prediction models, given the differences in Asian and Non-Asian population characteristics, but we also included several well-recognised or/and widely-used non-Asian derived prediction models for comparison.

## Methods

### Models for validation (Asian and non-Asian derived diabetes risk prediction models)

#### Literature search for Asian-derived T2D risk prediction models

A systematic literature search was performed in MEDLINE and EMBASE to identify Asian-derived T2D risk prediction models studies published until February 2022 (the literature search strategy is summarized in Additional file 1: Supplementary Text). The review was performed according to the PRISMA guideline by two independent reviewers (SX and QX, checklist in Additional file 1: Table S1) [6]. The process of defining the review question, study eligibility criteria, and data extraction was performed following the applicable guidance from a checklist for critical appraisal and data extraction for systemic review of prediction modelling studies (CHARMS) [7].

The main inclusion criteria for prediction model studies included: (1) Prognostic prediction model to predict future risk of T2D; (2) Model development was based on a prospective cohort; (3) The derivation populations (i.e., the population for model development) were Asian adults without diabetes at baseline; (4) Predictors were routinely-available clinical variables. The detailed inclusion criteria and exclusion criteria are listed in Additional file 1: Table S2.

#### Prespecified non-Asian derived T2D risk prediction models

The San Antonio model [8], Finnish diabetes risk model (FINDRISC) [9], Atherosclerosis Risk in Communities Model [10] and Framingham diabetes risk model [11] were included for validation because they are currently well-recognised or/and widely-used non-Asian derived diabetes risk prediction models. The STOP-NIDDM model was also included because it was built for people with IH [12]. These prespecified models met all the criteria listed in Additional file 1: Table S2, except for being derived in non-Asian adults.

The Asian and non-Asian derived T2D risk prediction models were divided into BASIC (non-invasive variables only) and EXTENDED models (plus laboratory variables). If there were several BASIC or EXTENDED models in one study, the one with the best reported performance was used for this validation study.

#### Assessment of risk of bias

We assessed the risk of bias of the included prediction model studies following the short form guidelines of the Prediction study Risk of Bias Assessment tool (PROBAST) [13]. This was done independently by two researchers (SX and QX). Disagreement was resolved through discussions with a third researcher.

### Validation populations (ACE, Luzhou, and TCLSIH cohorts)

#### Cohort profiles

The Acarbose Cardiovascular Evaluation (ACE) was a randomized, double-blind, placebo-controlled, event-driven, Phase IV superiority trial conducted in 176 outpatient clinics in China [14]. Eligible participants were aged 50 years or older with established coronary heart disease and IGT (confirmed by a 75 g oral glucose tolerance test [OGTT]). Between March 2009 and October 2015, 6522 eligible patients were randomized to acarbose 50 mg TID or matching placebo, and were followed until April 2017.

The Luzhou cohort was a prospective community-based cohort study which used a multistage cluster random sampling strategy to enroll residents aged 40 and older from five communities in Luzhou city of China. It was part of the Risk Evaluation of cAncers in Chinese diabeTic Individuals: a lONgitudinal (REACTION) study, a multicentre prospective observational study investigating the association between diabetes and the risk of cancer in mainland China [15]. A total of 10,007 residents were enrolled in 2011, who were revisited in 2014 and/or 2016.

The Tianjin Chronic Low-grade Systemic Inflammation and Health Cohort Study (TCLSIH) was a large prospective dynamic cohort study that randomly recruited participants during routine preventive examinations (annual physical examinations) at the Tianjin Medical University General Hospital-Health Management Centre. The TCLSIH mainly focused on the relationship between chronic low-grade systemic inflammation and the health status of a population living in Tianjin city of China [16]. Between 2007 and 2018, 42,521 participants were enrolled and followed annually.

#### Assessment and definition of glycemia

In the ACE study, fasting plasma glucose (FPG) was measured every 4 months and a 75 g OGTT was performed annually, with a confirmatory OGTT done if either of these tests suggested diabetes. In the TCLSIH and Luzhou cohorts, FPG, HbA_1c_ and 2-h plasma glucose (2HPG) from OGTT were measured at baseline and during subsequent revisits. Definitions of diabetes and IH in these three validation populations were all based on the 1999 World Health Organization diagnostic criteria [17]. Specifically, progression to diabetes was defined as an elevated FPG (≥ 7.0 mmol/L) and/or 2HPG (≥ 11.1 mmol/L), or a diagnosis of diabetes made by physicians, which in the ACE study would be further confirmed by the independent ACE Diabetes Adjudication Committee.

#### Inclusion of validation populations

Participants with IH at baseline and had information on diabetes status during follow-up were eligible for this validation study. Additionally, only the placebo group of the ACE study were considered for the validation analysis because acarbose has been shown to reduce the risk of diabetes [14].

### Statistical analysis and model validation

#### Missing data and missing predictors

There were less than 10% of missing values for most variables, except for prior hypertension (15%) and prior cardiovascular disease (17%) in the Luzhou cohort, and current alcohol drinking (15%) in the TCLSIH cohort. Missing values were imputed by multiple imputations (MICE package, R). We repeated the validation analyses among three cohorts using only complete cases of the requisite variables as a sensitivity analysis.

Information for most predictors were available in all three validation cohorts. When no information of predictors was available in the validation datasets, a fixed value for the “missing variables” (i.e., “0” for categorial variables and a fixed number for continuous variables) was used for validation analysis.

#### Predicted vs. observed risk

Comparing the predicted risk with the observed risk in the validation populations indicates whether the prediction model overestimates or underestimates actual risk. We calculated the predicted to observed risk ratio (P/O) with a 95% confidence interval to quantify this comparison. A P/O value equal to 1.0, or its 95% CI crossing 1.0, indicates that the predicted risk falls within the observed risk range, whereas P/O values less or more than 1.0 suggested that the model underestimates or overestimates the actual risk respectively.

As the prediction horizon of the models examined could differ from the median follow-up duration of the validation cohorts, predictions were standardized by dividing predicted risk was by the prediction horizon (years), multiplied by the actual median follow-up time (years) of the validation cohort. This is based on our assumption that the annual risk of progression to diabetes from IH does not vary over time, as seen in previous diabetes prevention trials [18].

#### Discrimination and calibration

Discrimination indicates the ability of a prediction model to separate those who develop diabetes from those who do not. We used the C-statistic to classify discrimination, as poor (0.5 to < 0.6), moderate (0.6 to < 0.7), good (0.7 to < 0.8), very good (0.8 to < 0.9) or excellent (≥ 0.9) [19].

Calibration measures how closely predicted outcomes agree with observed outcomes across groups of individuals. The overall calibration feature can be estimated by the Hosmer–Lemeshow test, with a good fit indicated by a *P*-value > 0.05.

#### Recalibration

Differences in the incidence rate of diabetes between the derivation populations and the validation populations would lead to significant deviations between the predicted risk (by the prediction models) and observed risk in the validation cohorts. Accordingly, we recalibrated each prediction model by adjusting the intercept (for logistic regression models) or the baseline survival function (for survival regression models). The recalibration process does not affect discrimination, so only P/O values and calibration were re-evaluated after recalibration.

#### Risk stratification for Chinese IH

The ability of risk stratification for Chinese IH was compared between the validated risk prediction models and the “Chinese IH risk stratification” strategy. The cut-off points for risk stratification using prediction models were based on annual diabetes risk as described previously (modest risk: 0–5%; moderate risk: 5–10%; high risk: > 10%) [20].

This external validation study was reported in compliance with the TRIPOD statement [21]. *P* < 0.05 was considered to be statistically significant. All statistical analyses were performed using R (version 4.1.2).

## Results

### Characteristics of the included models

The systematic literature review process is shown in Additional file 1: Figure S1. A total of 5173 records (MEDINLE n = 1810, EMBASE n = 3363) were identified through database searches. After removal of duplicates, 3736 records were assessed for eligibility, of which 44 Asian-derived (41 all-Asian and three part-Asian) T2D risk prediction model studies were selected [20, 22–64]. With the five prespecified non-Asian derived diabetes risk prediction model studies [8–12], 49 T2D risk prediction model studies were included in this validation study. However, majority of these studies were at high (n = 43, 87.8%) or unclear (n = 3, 6.1%) risk of bias, while only three studies (6.1%) were scored at low risk of bias (Table 1 and Additional file 1: Supplementary Text).

**Table 1:** Characteristics of the included risk prediction models for incident type 2 diabetes

No.	Author, year	Ethnicity	Derivation sample			Overall risk of bias^†^
			Glycemic categories	Diabetes cases/sample size	Follow-up duration (years)	
*All-Asian derived models*						
1	Aekplakorn, 2006 [22]	Asian (Thai)	Non-diabetes*	361/2677	12	High
2	Chien, 2009 [23]	Asian (Chinese)	Non-diabetes*	548/2960	10	High
3	Gao, 2009 [24]	Asian (Indian)	Non-diabetes*	511/3094	11	High
4	Sun, 2009 [25]	Asian (Chinese)	Non-diabetes*	902/20,551	3.2	High
5	Chuang, 2011 [26]	Asian (Chinese)	Non-diabetes*	1261/19,919	5.6	High
6	Liu, 2011 [27]	Asian (Chinese)	Non-diabetes*	304/1457	10	High
7	Doi, 2012 [28]	Asian (Japanese)	Non-diabetes*	286/1935	14	High
8	Heianza, 2012 [29]	Asian (Japanese)	Non-diabetes*	289/7654	5	High
9	Lim, 2012 [30]	Asian (Korean)	Non-diabetes*	436/6342	4	High
10	Xu, 2014 [31]	Asian (Chinese)	Non-diabetes*	836/16,043	5.2	High
11	Ye, 2014 [32]	Asian (Chinese)	Non-diabetes*	924/1912	6	High
12	Nanri, 2015 [33]	Asian (Japanese)	Non-diabetes*	1122/24,950	3	High
13	Liu, 2016 [34]	Asian (Chinese)	Non-diabetes*	215/1857	10.9	High
14	Wang, 2016 [35]	Asian (Chinese)	Non-diabetes*	4726/49,325	5.4	High
15	Zhang, 2016 [36]	Asian (Chinese)	Non-diabetes*	729/12,849	6	High
16	Miyakoshi, 2016 [37]	Asian (Japanese)	Non-diabetes*	138/2080	4.9	High
17	Chen, 2017 [38]	Asian (Chinese)	Non-diabetes*	387/28,251	4.2	High
18	Wen, 2017 [39]	Asian (Chinese)	Non-diabetes*	218/4132	6	High
19	Yokota, 2017 [40]	Asian (Japanese)	IH	252/2105	4.7	Unclear
20	Zhang, 2017 [41]	Asian (Chinese)	Non-diabetes*	702/15,758	6	High
21	Ha, 2018 [42]	Asian (Korean)	Non-diabetes*	37,678/359,349	10.8	High
22	Han, 2018 [43]	Asian (Chinese)	Non-diabetes*	1390/17,690	4	High
23	Hu, 2018 [44]	Asian (Japanese)	Non-diabetes*	2216/30,500	7	High
*Asian derived models*						
24	Ustulin, 2018 [45]	Asian (Korean)	IH	801/1162	4.0	High
25	Yastuya, 2018 [46]	Asian (Japanese)	Non-diabetes*	342/3540	12.2	High
26	Wang, 2019 [47]	Asian (Chinese)	Non-diabetes*	595/5557	3	High
27	Cai, 2020 [48]	Asian (Chinese)	Non-diabetes*	81/1273	3	High
28	Hu, 2020 [49]	Asian (Chinese)	Normoglycemia	171/4833	4.6	High
29	Lin, 2020 [50]	Asian (Chinese)	Non-diabetes*	466/21,844	3.1	High
30	Liu, 2020-1 [51]	Asian (Chinese)	Non-diabetes*	2623/43,404	6.83	Low
31	Liu, 2020-2 [52]	Asian (Chinese)	Non-diabetes*	2151/58,056	2.98	High
32	Ma, 2020 [53]	Asian (Chinese)	Non-diabetes*	256/10,807	6.0	High
33	Shao, 2020 [54]	Asian (Chinese)	Non-diabetes*	257/4498	10	High
34	Wang, 2020 [55]	Asian (Japanese)	Normoglycemia	275/8296	7.75	High
35	Wu, 2020 [56]	Asian (Chinese)	Non-diabetes*	155/16,219	2.66	Unclear
36	Cai, 2021-1 [57]	Asian (Japanese)	Normoglycemia	157/2058	5.1	High
37	Cai, 2021-2 [58]	Asian (Japanese)	Normoglycemia	154/9651	5.4	High
38	Li, 2021 [59]	Asian (Chinese)	Non-diabetes*	74/687	15	High
39	Liang, 2021 [60]	Asian (Chinese)	IH	145/1857	3	High
40	Wu, 2021 [61]	Asian (Chinese)	Non-diabetes*	145/7940	3	High
41	Xu, 2021 [62]	Asian (Chinese)	IGT	493/3105	5	High
*Part-Asian derived models*						
42	Chen, 2009 [63]	Caucasian, Asian, others	Non-diabetes*	362/6060	5	High
43	Bethel, 2013 [20]	Black, Caucasian, Asian, others	IGT	3254/9306	5	Low
44	Hippisley-Cox, 2018 [64]	Caucasian, Chinese, Asian, others	Non-diabetes*	178,314/8,186,705	3.9	Low
*Non-Asian derived models*						
45	Stern, 2002 [8]	Mexican Americans, non-Hispanic whites	Non-diabetes*	269/2903	7.5	High
46	Lindstrom, 2003 [9]	Caucasian	Non-diabetes*	182/4435	10	High
47	Schmidt, 2005 [10]	Caucasian, African Americans	Non-diabetes*	1292/7915	9	High
48	Wilson, 2007 [11]	Caucasian	Non-diabetes*	160/3140	7	Unclear
49	Tuomilehto, 2010 [12]	Caucasian	IGT	398/1160	2.5	High

*IH* Intermediate hyperglycemia, *IGT* Impaired glucose tolerance

^*^In the present study, glycemic category of non-diabetes includes normoglycemia and intermediate hyperglycemia

^†^The risk of bias of the included prediction model studies was assessed following the short form guidelines of the Prediction study Risk of Bias Assessment tool (PROBAST) [13]

These 49 risk prediction model studies comprised 21 BASIC and 46 EXTENDED models for validation, with prediction horizons varying from 2.5 to 20 years. Model performances reported by the original studies are summarized in Additional file 1: Table S3. Their predictors varied from 4 to 17 items in BASIC models (age, body mass index, and blood pressure were the most commonly used non-invasive predictors), and 3 to 17 items in EXTENDED models (FPG, triglycerides, and HbA_1C_ were most commonly-used laboratory predictors) (Additional file 1: Tables S4, S5).

### Characteristics of the validation populations

A total of 3241, 1333, and 1702 IH participants of the ACE, Luzhou, and TCLSIH cohorts, respectively, were eligible for the main validation (Additional file 1: Figure S2). Their baseline characteristics are summarized in Additional file 1: Table S6. Among them, 509 (15.7%), 260 (19.5%), and 396 (23.3%) of the three cohorts, respectively, developed diabetes over a median follow-up of 5.0, 3.0, and 3.0 years.

### External validation of the included models

**Fig. 1:**
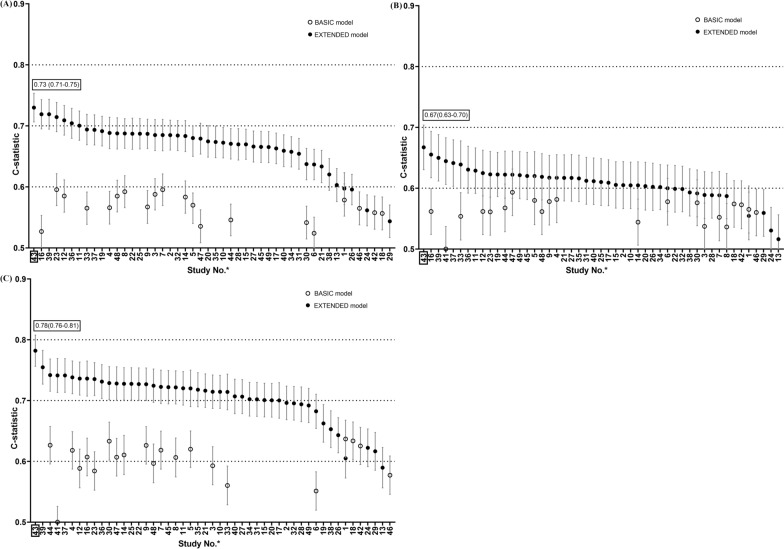
Discrimination of the included models in the **A** ACE, **B** Luzhou, and **C** TCLSIH cohorts. *: The corresponding specific study of each Study No. can be found in Table 1. The Study No. was ordered by the value of C-statistics. The Study No. marked with a square indicated that its prediction model had the best discrimination in the validation cohort, with a label of its C-statistic in the upper left corner of the figure

In the ACE, Luzhou, and TCLSIH cohorts, BASIC models showed poor-to-moderate discrimination with C-statistics 0.52–0.60, 0.50–0.59, and 0.50–0.64, respectively. EXTENDED models showed poor-to-acceptable discrimination (C-statistic: 0.54–0.73, 0.52–0.67, and 0.59–0.78, respectively). The EXTENDED model of the Nateglinide and Valsartan in Impaired Glucose Tolerance Outcomes Research (*NAVIGATOR*) study (study 43) had the best discrimination in the three cohorts with C-statistics of 0.73, 0.67 and 0.78, respectively (Fig. 1 and Additional file 1: Tables S7, S8).

Fifteen BASIC and 40 EXTENDED models had full information (e.g., intercept or baseline survival function of regression equation, or detailed scoring system) for calculating predicted risk. They all showed poor calibration based on the Hosmer–Lemeshow test (*P* < 0.05). The majority of the 15 BASIC models underestimated (P/O: 0.11–0.79, 0.03–0.48, and 0.04–0.44) the diabetes risk among the ACE (6/14), Luzhou (14/15), and TCLSIH (14/15) cohorts. Most of the 40 EXTENDED models also underestimated (P/O: 0.08–0.87 and 0.08–0.90) the diabetes risk in the Luzhou (26/40) and TCLSIH cohorts (31/40), while most of them overestimated (P/O: 1.12–5.45) the diabetes risk in the ACE cohorts (27/39) (Additional file 1: Tables S7, S8).

Most of the recalibrated models showed improved calibration but significant deviations between the observed and predicted risk by them still existed. The recalibrated BASIC (P/O: 0.93–1.54, 0.92–1.42, and 0.87–1.43) and EXTENDED models (P/O: 0.92–5.76, 0.88–4.69, and 0.88–3.99) from modestly to severely overpredicted the diabetes risk in the ACE, Luzhou, and TCLSIH cohorts (Additional file 1: Tables S7, S8).

When broadening the validation samples to non-diabetic participants (i.e., including normoglycemia and IH) in the Luzhou and TCLSIH cohorts (Additional file 1: Tables S9, S10), similar tendencies were observed but most of the models showed slightly higher discrimination (C-statistic: 0.51–0.72 and 0.55–0.89 respectively). Sensitivity analyses revealed overall similar results when using complete cases only (Additional file 1: Tables S11, S12).

### Risk stratification of IH

**Fig. 2:**
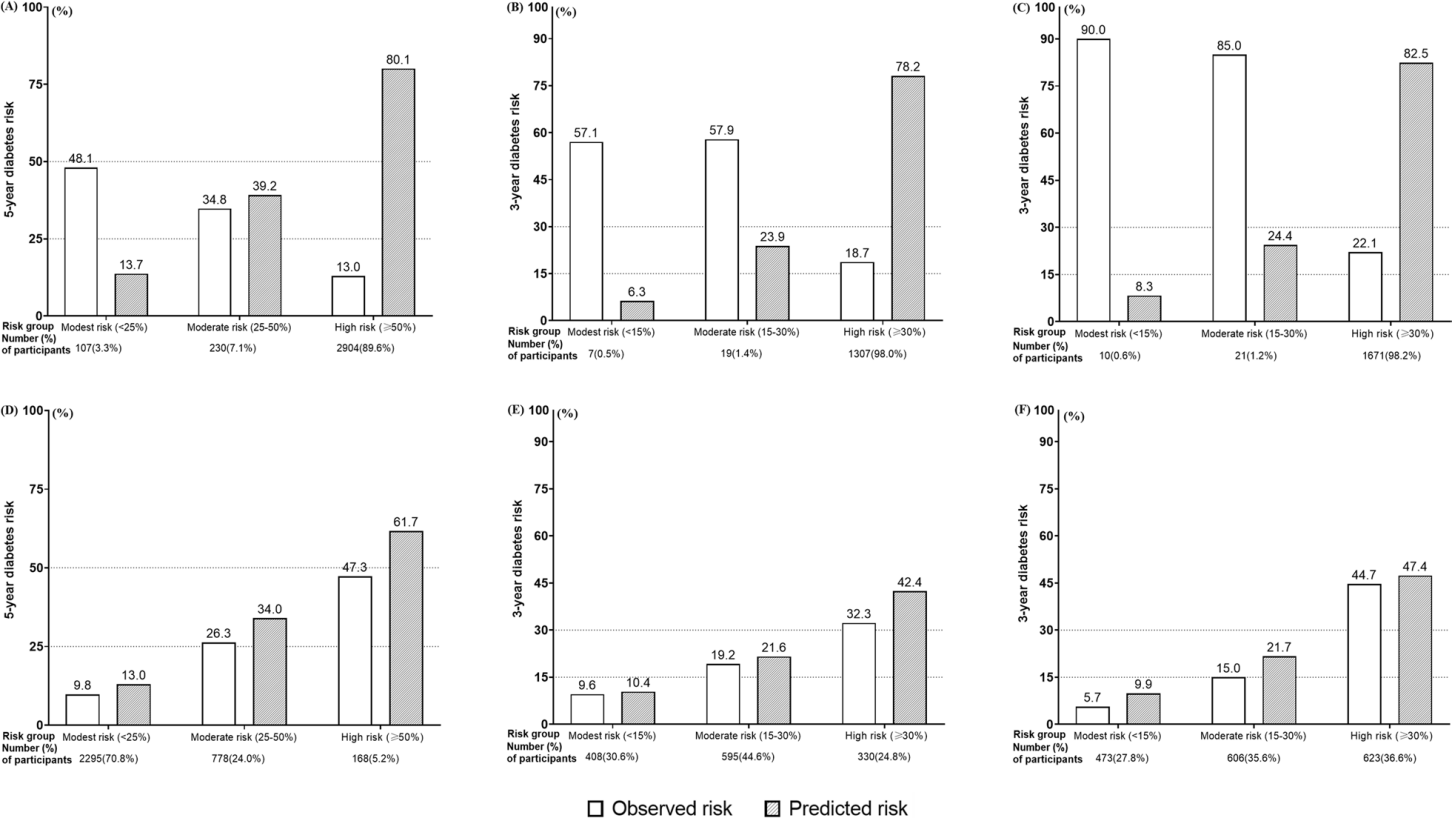
Predicted (vs. observed) diabetes risk of the original *NAVIGATOR* model in three risk classes of the **A** ACE, **B** Luzhou, and **C** TCLSIH cohorts, and the recalibrated *NAVIGATOR* model in **D** ACE, **E** Luzhou, and **F** TCLSIH cohorts

**Fig. 3:**
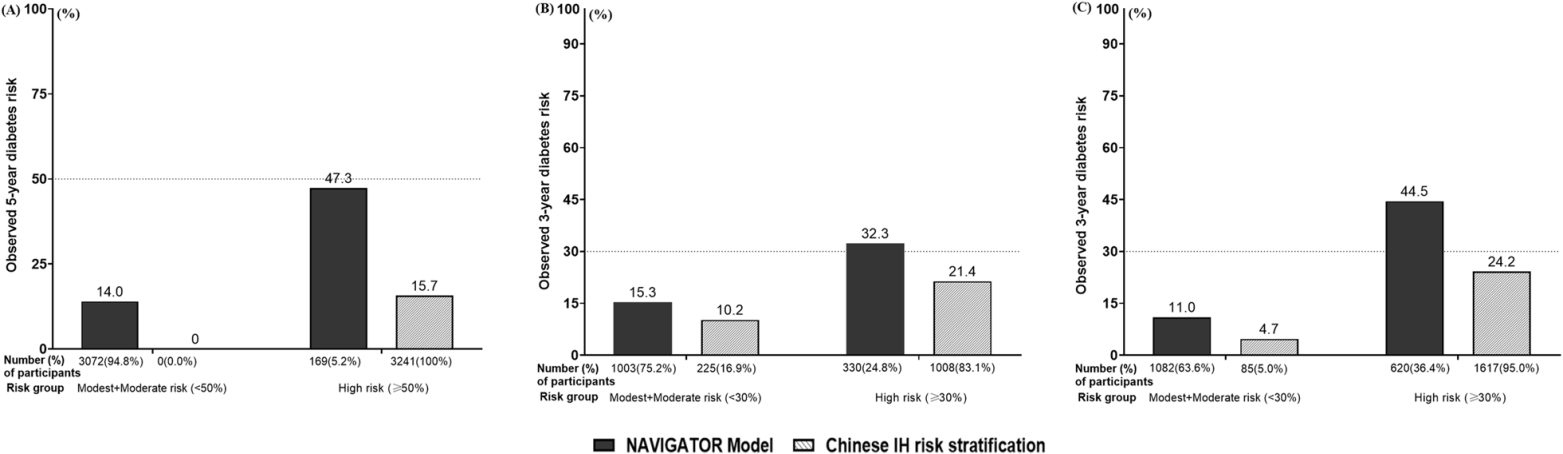
Risk stratification for the **A** ACE, **B** Luzhou, and **C** TCLSIH cohorts

The majority (89.6%, 98.0% and 98.2% of IH participants of the ACE, Luzhou, and TCLSIH cohorts) were classified as high risk by the *NAVIGATOR* model. Obvious deviations between observed risks and predicted risks by the original *NAVIGATOR* model were seen among three cohorts (Fig. 2A–C). After recalibration, the deviations were significantly improved but overprediction was noted across all risk groups (Fig. 2D–F). Compared with the recalibrated *NAVIGATOR* model, the “Chinese IH risk stratification” strategy tended to misclassify the individuals of modest or moderate risk into high risk among three cohorts (Fig. 3).

## Discussion

We conducted an external validation of 21 BASIC and 46 EXTENDED T2D risk prediction models in three independent prospective Chinese cohorts of people with IH. We found that BASIC models to predict T2D did not exhibit good discrimination or calibration while several EXTENDED models had acceptable discrimination but poor calibration. Most of the recalibrated models showed better calibration but still modestly to severely overestimated the diabetes risk in three populations.

People with IH are at high risk of diabetes development. It has been suggested that all people with IH are encouraged to practice appropriate lifestyle modifications while those at higher absolute risk may benefit from more intensive lifestyle modification and evidence-based preventive medications [20]. Therefore, knowledge of the future absolute risk of T2D is critical to inform the choice of intensity of the preventive intervention needed.

Thus, we conducted this validation study among three independent Chinese IH populations of different study settings. The Luzhou cohort is community-based population, while the TCLSIH and ACE cohorts are a health check-up-based population and a randomized intervention trial population, respectively. Compared with the Luzhou and ACE cohorts, the IH participants of the TCLSIH cohort were youngest but had the worst metabolic phenotypes (more likely to smoke, take alcohol, and have highest obese measurements and worst lipid profiles). The TCLSIH cohort had the highest annual diabetes incidence rate (23.3% vs. 19.5% and 15.7% over a median follow-up of 3.0, 3.0, and 5.0 years, respectively). This is consistent with previous findings showing that the age of IH onset is inversely associated with future diabetes progression risk [20, 62].

The majority of the included T2D risk prediction model studies showed an overall high or unclear risk of bias, which indicated that their predictive performance when used in practice is probably lower than that reported. This is consistent with our findings at validation.

The clinical usefulness of a model depends largely on its discrimination. Our results showed that all of the BASIC models did not have good discrimination in three Chinese IH cohorts, whilst several EXTENDED models did. In contrast, our validation results and previous validation studies [65] in non-diabetic participants (i.e., including normoglycemia and IH) showed that BASIC models could help identify individuals at high diabetes risk among non-diabetic population. These findings suggest that incorporating non-invasive information to predict diabetes risk is feasible to assess the risk of diabetes but not sufficient for the people with IH. Among the EXTENDED models, the *NAVIGATOR* model was one of the few models containing three glycaemic measurements (FPG, 2HPG and HbA_1C_) and at low risk of bias, which had the best discrimination in three validation cohorts. Previous findings of hyperglycaemia are obviously important predictors among the routinely-available clinical variables for predicting diabetes risk [1], since diabetes is a disease with slow progress from IH to diabetes. Therefore, among the existing models to predict the incident risk of T2D, the *NAVIGATOR* model is presumed to have the best discriminative ability for Chinese IH populations when FPG, 2HPG and HbA_1C_ values are available.

Calibration is also an essential requirement when the aim of using a prediction model is to inform decision-making in clinical practice. Our results showed that nearly all BASIC models and several EXTENDED models underestimated the actual diabetes risk in three validation populations. After recalibration (adjusting for the differences in the incidence of diabetes between the development populations and our validation populations), all models showed better calibration but were overall overpredicting the actual diabetes risk. It can be seen in the recalibrated *NAVIGATOR* model that this overestimation occurred in all risk groups. This overestimation may induce an unnecessary burden of overtreatment for individuals at actual low risk.

As for risk stratification, the “Chinese IH risk stratification” seemed an unsuitable strategy for risk stratification to guide primary prevention measures for Chinese IH populations. The fact is that the majority of IH individuals have at least one of the specified risk factors, meaning that they are very likely to be classified into the high-risk group. As seen in this study, many people with IH were misclassified into a higher risk category when using the “Chinese IH risk stratification” than using the recalibrated *NAVIGATOR* model. That is, many people with IH were up-classified as high risk by the “Chinese IH risk stratification”. The clinical implication of this up-classification was that it also increased the treatment burden for individuals at actual low risk.

In this study, we comprehensively validated the performances of the existing Asian and non-Asian derived models to predict the risk of incident T2D in three independent Chinese IH cohorts. Due to the large sample size, prospective longitudinal cohort design and contemporary nature of three validation cohorts, our findings were stable and generalizable. However, our study has some limitations. Firstly, glycemia was assessed more frequently in the ACE (annual OGTT and a confirmatory OGTT if necessary) and TCLSIH (annual OGTT) cohorts than in the Luzhou (OGTT only once at follow-up end) cohort. This might have led to under ascertainment of diabetes incidence (e.g., false-negative cases) to some extent, which resulted in underestimating the C-statistics in the Luzhou cohort and influencing calibration as we found in our validation results. Secondly, some validation datasets did not involve the collection of some parameters such as physical activity, dietary habits and education, which may have limited the performance of some validated models. However, most of the required variables were available in three datasets. Thus, it is unlikely that this influenced our results to a large extent. Similarly, missing data were only limited for a few participants, and this was handled by multiple imputations. We also conducted complete cases analyses, which yielded similar results to support our findings. Thirdly, while IH participants of the ACE cohort all had previous cardiovascular disease (CVD), IH participants of the Luzhou and TCLSIH cohorts had only a few people with prior CVD (5.6% and 9.8%, respectively). Due to the limited sample size of people with CVD in these two cohorts, we are unable to further explore whether the performance of the prediction models differed by CVD status in these cohorts. Fourthly, for models with different prediction horizons from the median follow-up duration of our validation cohorts, the predicted risks were projected based on the assumption that annual risk of incident T2D is equal, as seen in previous diabetes prevention trials [18]. But this may still to some extent influence our evaluation.

Generally speaking, our systematic review and external validation study indicated that the vast majority of published T2D models were not built with a robust modelling method and had poor external validity in Chinse people with IH. This implies that researchers should direct their efforts to help improve the generalizability of T2D models in the future, such as by applying a robust modelling method (e.g., select the representative derivation populations, handle missing data appropriately, and correct for model overfitting/optimism), and transparently reporting the models following the TRIPOD statement guideline which has been developed to support authors writing reports describing the development, validation or updating of prediction models. Furthermore, we encourage external validation research on the existing T2D models to understand their external validity on independent data, so as to know whether they can be effectively put into practice in a target population.

## Conclusions

For Chinese people with IH, BASIC models to predict T2D did not exhibit good discrimination or calibration. Several EXTENDED models performed better, but a robust Chinese diabetes risk prediction tool in people with IH remains an unmet need. To use these models to inform decision-making in clinical practice, in particular calibration needs to be further improved.

## Supplementary Information

**Additional file 1.** Supplementary Text. **Figure S1.** The literature review process of searching Asian-derived type 2 diabetes risk prediction models. **Figure S2.** Flowchart of the study population selection process of the ACE, Luzhou, and TCLSIH cohorts. **Table S1.** PRISMA checklist for reporting systematic review. **Table S2.** Checklist for critical appraisal and data extraction for systemic review of prediction modelling studies (CHARMS). **Table S3.** The performance of the included BASIC and EXTENDED models reported in their original studies. **Table S4.** Predictors included in the BASIC models (N = 21). **Table S5.** Predictors included in the EXTENDED models (N = 46). **Table S6.** Baseline characteristics of the participants with intermediate hyperglycemia at baseline of the ACE, Luzhou, and TCLSIH cohorts. **Table S7.** The validation results of the included BASIC models in intermediate hyperglycemia participants of the ACE, Luzhou, and TCLSIH cohorts. **Table S8.** The validation results of the included EXTENDED models in intermediate hyperglycemia participants of the ACE, Luzhou, and TCLSIH cohorts. **Table S9.** The validation results of the included BASIC models in non-diabetic participants of the Luzhou and TCLSIH cohorts. **Table S10.** The validation results of the included EXTENDED models in non-diabetic participants of the Luzhou and TCLSIH cohorts. **Table S11.** The validation results of the included BASIC models in intermediate hyperglycemia participants of the ACE, Luzhou, and TCLSIH cohorts when using complete cases for analysis. **Table S12.** The validation results of the included EXTENDED models in intermediate hyperglycemia participants of the ACE, Luzhou, and TCLSIH cohorts when using complete cases for analysis.

## Data Availability

Requests for data access and proposals for analyses of ACE can be submitted to the ACE Publications Committee using instructions found at: https://www.dtu.ox.ac.uk/ACE/. The datasets of the Luzhou and TCLSIH cohorts are available from the corresponding author on reasonable request.

## Abbreviations

ACE: Acarbose Cardiovascular Evaluation
CHARMS: Checklist for critical appraisal and data extraction for systemic review of prediction modelling studies
FPG: Fasting plasma glucose
2HPG: 2-H plasma glucose
IFG: Impaired fasting glucose
IGT: Impaired glucose tolerance
IH: Intermediate hyperglycemia
NAVIGATOR: Nateglinide and Valsartan in Impaired Glucose Tolerance Outcomes Research
OGTT: Oral glucose tolerance test
REACTION: Risk Evaluation of cAncers in Chinese diabeTic Individuals: a lONgitudinal
T2D: Type 2 diabetes
PROBAST: Prediction study Risk of Bias Assessment tool
TCLSIH: Tianjin Chronic Low-grade Systemic Inflammation and Health Cohort Study
