# Supplementary material for: Risk prediction models for incident type 2 diabetes in Chinese people with intermediate hyperglycemia: a systematic literature review and external validation study
Source: Cardiovasc Diabetol. 2022 Sep 13;21:182. doi: 10.1186/s12933-022-01622-5 (PMC9472437; doi:10.1186/s12933-022-01622-5)
Supplement: Supplementary file 1 — Additional file 1. Supplementary Text. Figure S1. The literature review process of searching Asian-derived type 2 diabetes risk prediction models. Figure S2. Flowchart of the study population selection process of the ACE, Luzhou, and TCLSIH cohorts. Table S1. PRISMA checklist for reporting systematic review. Table S2. Checklist for critical appraisal and data extraction for systemic review of prediction modelling studies (CHARMS). Table S3. The performance of the included BASIC and EXTENDED models reported in their original studies. Table S4. Predictors included in the BASIC models (N = 21). Table S5. Predictors included in the EXTENDED models (N = 46). Table S6. Baseline characteristics of the participants with intermediate hyperglycemia at baseline of the ACE, Luzhou, and TCLSIH cohorts. Table S7. The validation results of the included BASIC models in intermediate hyperglycemia participants of the ACE, Luzhou, and TCLSIH cohorts. Table S8. The validation results of the included EXTENDED models in intermediate hyperglycemia participants of the ACE, Luzhou, and TCLSIH cohorts. Table S9. The validation results of the included BASIC models in non-diabetic participants of the Luzhou and TCLSIH cohorts. Table S10. The validation results of the included EXTENDED models in non-diabetic participants of the Luzhou and TCLSIH cohorts. Table S11. The validation results of the included BASIC models in intermediate hyperglycemia participants of the ACE, Luzhou, and TCLSIH cohorts when using complete cases for analysis. Table S12. The validation results of the included EXTENDED models in intermediate hyperglycemia participants of the ACE, Luzhou, and TCLSIH cohorts when using complete cases for analysis. [file 12933_2022_1622_MOESM1_ESM.docx]

**Risk prediction models for incident type 2 diabetes in Chinese people with intermediate hyperglycemia: a systematic literature review and external validation study**

Shishi Xu, Ruth Coleman, Qin Wan, Yeqing Gu, Ge Meng, Kun Song, Zumin Shi, Qian Xie, Jaakko Tuomilehto, Rury R. Holman, Kaijun Niu, Nanwei Tong

**Additional file 1**

**Table of contents**

[**Supplementary Text** 2](#_Toc112663081)

[**Figure S1** The literature review process of searching Asian-derived type 2 diabetes risk prediction models. 3](#_Toc112663082)

[**Figure S2** Flowchart of the study population selection process of the ACE, Luzhou, and TCLSIH cohorts. 4](#_Toc112663083)

[**Table S1** PRISMA checklist for reporting systematic review 5](#_Toc112663084)

[**Table S2** Checklist for critical appraisal and data extraction for systemic review of prediction modelling studies (CHARMS). 9](#_Toc112663085)

[**Table S3** The performance of the included BASIC and EXTENDED models reported in their original studies. 10](#_Toc112663086)

[**Table S4** Predictors included in the BASIC models (N=21). 16](#_Toc112663087)

[**Table S5** Predictors included in the EXTENDED models (N=46). 17](#_Toc112663088)

[**Table S6** Baseline characteristics of the participants with intermediate hyperglycemia at baseline of the ACE, Luzhou, and TCLSIH cohorts. 20](#_Toc112663089)

[**Table S7** The validation results of the included BASIC models in intermediate hyperglycemia participants of the ACE, Luzhou, and TCLSIH cohorts. 21](#_Toc112663090)

[**Table S8** The validation results of the included EXTENDED models in intermediate hyperglycemia participants of the ACE, Luzhou, and TCLSIH cohorts. 23](#_Toc112663091)

[**Table S9** The validation results of the included BASIC models in non-diabetic participants of the Luzhou and TCLSIH cohorts. 27](#_Toc112663092)

[**Table S10** The validation results of the included EXTENDED models in non-diabetic participants of the Luzhou and TCLSIH cohorts. 28](#_Toc112663093)

[**Table S11** The validation results of the included BASIC models in intermediate hyperglycemia participants of the ACE, Luzhou, and TCLSIH cohorts when using complete cases for analysis. 30](#_Toc112663094)

[**Table S12** The validation results of the included EXTENDED models in intermediate hyperglycemia participants of the ACE, Luzhou, and TCLSIH cohorts when using complete cases for analysis. 32](#_Toc112663095)

**Supplementary Text**

1. **Literature search strategy for published Asian-derived type 2 diabetes risk prediction models**

The search strategy for MEDLINE was: (((Risk Assessment.sh.) or (risk score or risk model or risk prediction or risk equation or predict$ equation or scor$ system or algorithm or predict$ model or predict$).ti,ab.) AND (Incidence.sh.)) AND (Diabetes Mellitus, Type 2.sh.). The search strategy for EMBASE was: (((Risk Assessment.sh.) or (risk score or risk model or risk prediction or risk equation or predict$ equation or scor$ system or algorithm or predict$ model or predict$).ti,ab.) AND (Incidence.sh.)) AND (non insulin dependent diabetes mellitus.sh.). We initially searched databases in July 2019 and update them in February 2022.

1. **Summarized text of the included 49 T2D risk prediction model studies**

**Risk of bias:** There were 43 studies being assessed at high risk of bias, 3 at unclear risk of bias, and only 3 studies at low risk of bias. The majority of these studies scored a high or unclear risk of bias because of their analysis, with most models not informing or performing on the correct handling of missing data or correction for overfitting/optimism in model performance.

**Presentation formats:** The models included in this study were presenting in four formats: 1) Nomograms (n=14); 2) Scoring chart and similar derivations (n=23); 3) Regression formula (n=10); 4) Risk score and Regression formula (n=2).

**Validation type:** There were only 12 studies that had both internal and external validation in their original studies, while the other 37 studies had internal validation only. Worse still, there were 21 studies that applied the inappropriate “random split-sample method”.

**Calibration reporting:** There were only 18 studies that had calibration assessment (Calibration plot or Hosmer-Lemeshow test) in their original studies, while the other 31 studies had no information about calibration.


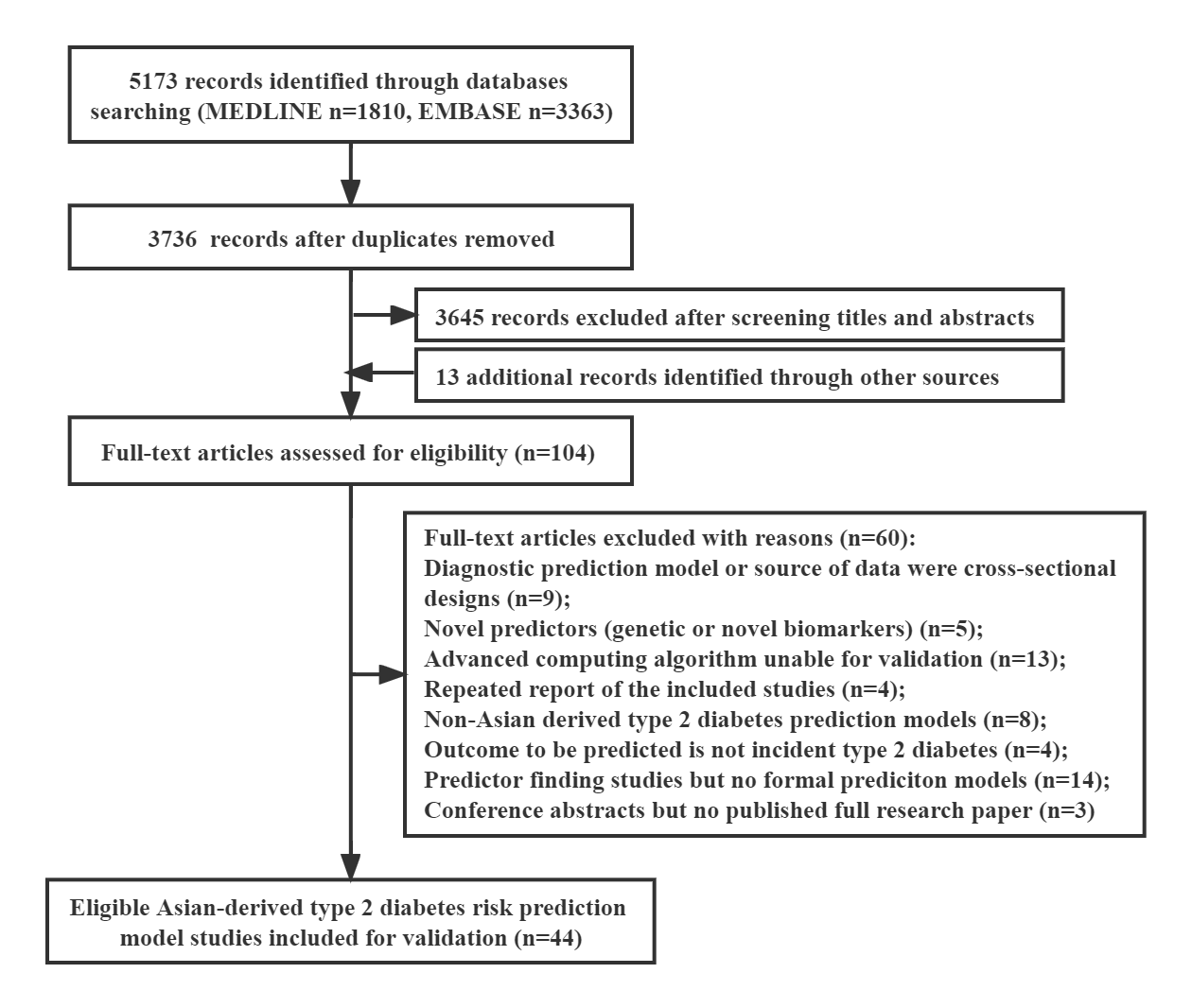


**Figure S1** The literature review process of searching Asian-derived type 2 diabetes risk prediction models.


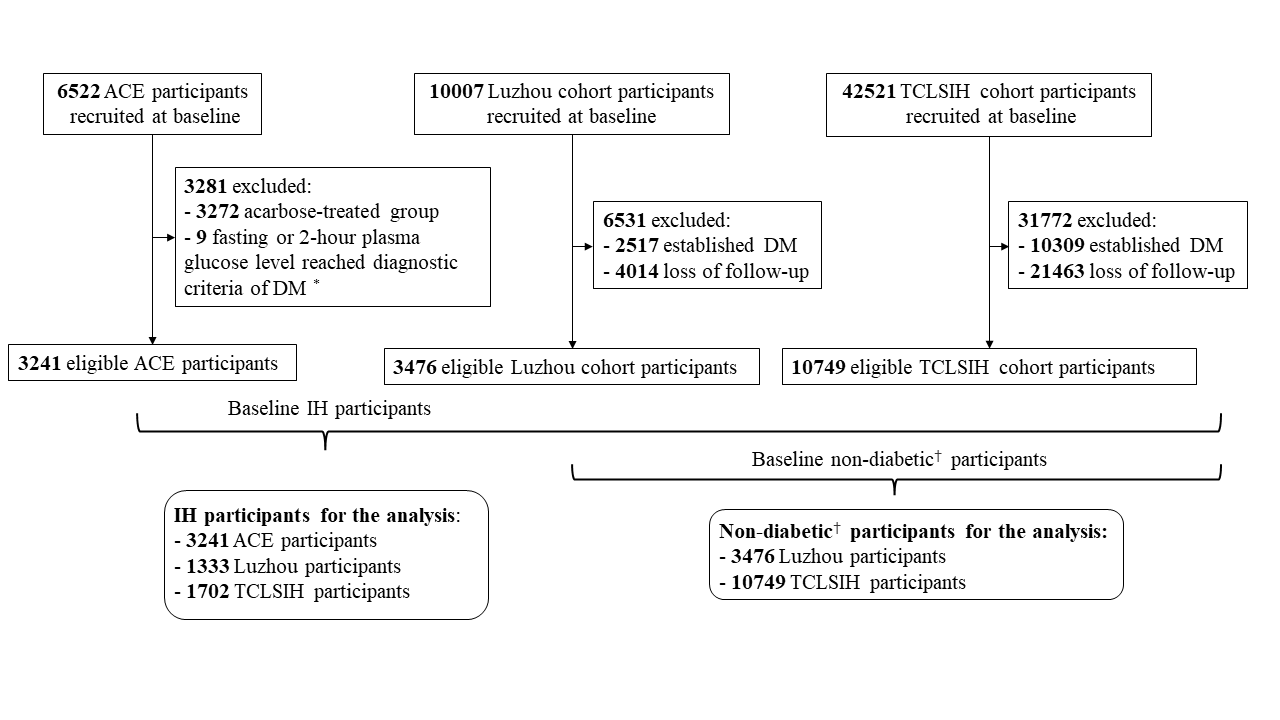


**Figure S2** Flowchart of the study population selection process of the ACE, Luzhou, and TCLSIH cohorts.

*: Some participants had fasting or 2-hour plasma glucose level which reached diagnostic criteria of diabetes mellitus because these values used were taken from the screening visit rather than the revisit after a 4-week run-in period before randomization. ^†^: In the present study, non-diabetic participants include those with normoglycemia and intermediate hyperglycemia. IH: Intermediate hyperglycemia. DM: Diabetes mellitus.

**Table S1** PRISMA checklist for reporting systematic review

| **Section and Topic** | **Item #** | **Checklist item** | **Page** |
| --- | --- | --- | --- |
| **TITLE** | | |  |
| Title | 1 | Identify the report as a systematic review. | 1 |
| **ABSTRACT** | | |  |
| Abstract | 2 | See the PRISMA 2020 for Abstracts checklist. | 3 |
| **INTRODUCTION** | | |  |
| Rationale | 3 | Describe the rationale for the review in the context of existing knowledge. | 5 |
| Objectives | 4 | Provide an explicit statement of the objective(s) or question(s) the review addresses. | 5 |
| **METHODS** | | |  |
| Eligibility criteria | 5 | Specify the inclusion and exclusion criteria for the review and how studies were grouped for the syntheses. | 6 |
| Information sources | 6 | Specify all databases, registers, websites, organisations, reference lists and other sources searched or consulted to identify studies. Specify the date when each source was last searched or consulted. | 6 |
| Search strategy | 7 | Present the full search strategies for all databases, registers and websites, including any filters and limits used. | 6;Supplemen-tary Text |
| Selection process | 8 | Specify the methods used to decide whether a study met the inclusion criteria of the review, including how many reviewers screened each record and each report retrieved, whether they worked independently, and if applicable, details of automation tools used in the process. | 6 |
| Data collection process | 9 | Specify the methods used to collect data from reports, including how many reviewers collected data from each report, whether they worked independently, any processes for obtaining or confirming data from study investigators, and if applicable, details of automation tools used in the process. | 6 |
| Data items | 10a | List and define all outcomes for which data were sought. Specify whether all results that were compatible with each outcome domain in each study were sought (e.g. for all measures, time points, analyses), and if not, the methods used to decide which results to collect. | 6-7; Table S5 |
|  | 10b | List and define all other variables for which data were sought (e.g. participant and intervention characteristics, funding sources). Describe any assumptions made about any missing or unclear information. | 6-7; Table S6-S7 |
| Study risk of bias assessment | 11 | Specify the methods used to assess risk of bias in the included studies, including details of the tool(s) used, how many reviewers assessed each study and whether they worked independently, and if applicable, details of automation tools used in the process. | 7 |
| Effect measures | 12 | Specify for each outcome the effect measure(s) (e.g. risk ratio, mean difference) used in the synthesis or presentation of results. | NA |
| Synthesis methods | 13a | Describe the processes used to decide which studies were eligible for each synthesis (e.g. tabulating the study intervention characteristics and comparing against the planned groups for each synthesis (item #5)). | NA |
|  | 13b | Describe any methods required to prepare the data for presentation or synthesis, such as handling of missing summary statistics, or data conversions. | NA |
|  | 13c | Describe any methods used to tabulate or visually display results of individual studies and syntheses. | NA |
|  | 13d | Describe any methods used to synthesize results and provide a rationale for the choice(s). If meta-analysis was performed, describe the model(s), method(s) to identify the presence and extent of statistical heterogeneity, and software package(s) used. | NA |
|  | 13e | Describe any methods used to explore possible causes of heterogeneity among study results (e.g. subgroup analysis, meta-regression). | NA |
|  | 13f | Describe any sensitivity analyses conducted to assess robustness of the synthesized results. | NA |
| Reporting bias assessment | 14 | Describe any methods used to assess risk of bias due to missing results in a synthesis (arising from reporting biases). | NA |
| Certainty assessment | 15 | Describe any methods used to assess certainty (or confidence) in the body of evidence for an outcome. | NA |
| **RESULTS** | | |  |
| Study selection | 16a | Describe the results of the search and selection process, from the number of records identified in the search to the number of studies included in the review, ideally using a flow diagram. | 12; Figure S1 |
|  | 16b | Cite studies that might appear to meet the inclusion criteria, but which were excluded, and explain why they were excluded. | Figure S1 |
| Study characteristics | 17 | Cite each included study and present its characteristics. | 13; Table 1 |
| Risk of bias in studies | 18 | Present assessments of risk of bias for each included study. | 13; Table 1 |
| Results of individual studies | 19 | For all outcomes, present, for each study: (a) summary statistics for each group (where appropriate) and (b) an effect estimate and its precision (e.g. confidence/credible interval), ideally using structured tables or plots. | Table S5 |
| Results of syntheses | 20a | For each synthesis, briefly summarise the characteristics and risk of bias among contributing studies. | NA |
|  | 20b | Present results of all statistical syntheses conducted. If meta-analysis was done, present for each the summary estimate and its precision (e.g. confidence/credible interval) and measures of statistical heterogeneity. If comparing groups, describe the direction of the effect. | NA |
|  | 20c | Present results of all investigations of possible causes of heterogeneity among study results. | NA |
|  | 20d | Present results of all sensitivity analyses conducted to assess the robustness of the synthesized results. | NA |
| Reporting biases | 21 | Present assessments of risk of bias due to missing results (arising from reporting biases) for each synthesis assessed. | NA |
| Certainty of evidence | 22 | Present assessments of certainty (or confidence) in the body of evidence for each outcome assessed. | NA |
| **DISCUSSION** | | |  |
| Discussion | 23a | Provide a general interpretation of the results in the context of other evidence. | 17 |
|  | 23b | Discuss any limitations of the evidence included in the review. | 20-21 |
|  | 23c | Discuss any limitations of the review processes used. | 20-21 |
|  | 23d | Discuss implications of the results for practice, policy, and future research. | 19-22 |
| **OTHER INFORMATION** | | |  |
| Registration and protocol | 24a | Provide registration information for the review, including register name and registration number, or state that the review was not registered. | Not registered |
|  | 24b | Indicate where the review protocol can be accessed, or state that a protocol was not prepared. | Supplementary Text |
|  | 24c | Describe and explain any amendments to information provided at registration or in the protocol. | Supplementary Text |
| Support | 25 | Describe sources of financial or non-financial support for the review, and the role of the funders or sponsors in the review. | 24 |
| Competing interests | 26 | Declare any competing interests of review authors. | 23-24 |
| Availability of data, code and other materials | 27 | Report which of the following are publicly available and where they can be found: template data collection forms; data extracted from included studies; data used for all analyses; analytic code; any other materials used in the review. | 23 |

NA: not applicable

**Table S2** Checklist for critical appraisal and data extraction for systemic review of prediction modelling studies (CHARMS).

| **Key items** | **Review aim** | **Inclusion criteria** | **Exclusion criteria** |
| --- | --- | --- | --- |
| **Prognostic versus diagnostic prediction model** | Prognostic prediction model | To predict future risk of T2D incidence | To detect prevalent/undiagnosed T2D |
| **Intended scope of the review** | Models to inform physician’s therapeutic decision | … | … |
| **Type of prediction modelling studies** | … | Prediction model development study, with or without external validation in independent data | External model validation study |
| **Target population to whom the prediction model applies** | Asian adults without diabetes at baseline | The derivation samples were completely Asian adults or partly Asian adults. | The derivation samples were children or adolescents, or completely non-Asians. |
| **Outcome to be predicted** | New-onset T2D | Incidence of T2D | Incidence of GDM or T1D |
| **Time span of prediction** | No limit | … | … |
| **Intended moment of using the model** | To help patients without diabetes predict their future T2D risk | … | … |
| **Source of data** | … | Longitudinal design study | Cross-sectional study |
| **Predictors** | … | Conventional and clinical-available factors (i.e., demographical characteristics, or routinely-available laboratory variables) | Novel predictors that were not routinely available, such as genetic factors or novel biomarker |
| **Modeling method** | Enable models to present as an equation or a scoring algorithm that can be used for further validation | Traditional multivariable logistic regression or survival models and their derivatives (simplified risk scores and risk calculators) | Advanced modeling methods such as hybrid prediction model, neural network, or machine learning |

T1D: Type 1 diabetes. T2D: Type 2 diabetes. GDM: Gestational diabetes mellitus.

**T****able S3** The performance of the included BASIC and EXTENDED models reported in their original studies.

| **Study No.** | **Prediction horizon** | **Modeling method** | **Presentation formats** | **Validation type** | **BASIC Model** | | **EXTENDED Model** | |
| --- | --- | --- | --- | --- | --- | --- | --- | --- |
|  |  |  |  |  | **C-Statistic** | **Calibration** | **C-Statistic** | **Calibration** |
| 1 | 12 years? | Logistic | Risk score (without corresponding absolute diabetes risk) | Internal validation | 0.74 (0.71-0.78) | Good^*^ | 0.79 (0.76-0.82) | Good^*^ |
|  |  |  |  | External validation (Geographical validation) | 0.75 (0.71-0.80) | NI | 0.81 | NI |
| 2 | 10 years | Cox | Score chart | Internal validation only | … | … | 0.702 (0.676-0.727) | NI |
| 3 | 11 years | Cox | Figure with absolute diabetes risk | Internal validation only (random split-sample) | 0.62 (0.56-0.68)^M^ 0.64 (0.59-0.69)^F^ | NI | 0.70 (0.64-0.75)^M^ 0.71 (0.66-0.76)^F^ | NI |
| 4 | 5 years | Cox | Regression equation | Internal validation only (random split-sample) | 0.751 | NI | 0.848 | Good^*^ |
| 5 | 10 years | Cox | Risk score | Internal validation only (random split-sample) | 0.761 (0.731-0.785) | NI | 0.835 (0.814-0.857) | NI |
| 6 | 10 years | Logistic | Risk score (without corresponding absolute diabetes risk) | Internal validation | 0.703 (0.670-0.737) | NI | 0.734 (0.702-0.766) | NI |
|  |  |  |  | External validation (Temporal validation) | 0.731 (0.659-0.804) | NI | 0.759 (0.686-0.831) | NI |
| 7 | 10 years | Cox | Risk score | Internal validation | 0.700 (0.677-0.732) | Good^*^ | 0.772 (0.741-0.802) | Good^*^ |
|  |  |  |  | External validation (Temporal validation) | 0.691 (0.633-0.749) | NI | 0.777 (0.727-0.827) | NI |

(continued)

| **Study No.** | **Prediction horizon** | **Modeling method** | **Presentation formats** | **Validation type** | **BASIC Model** | | **EXTENDED Model** | |
| --- | --- | --- | --- | --- | --- | --- | --- | --- |
|  |  |  |  |  | **C-Statistic** | **Calibration** | **C-Statistic** | **Calibration** |
| 8 | 5 years | Logistic | Score chart | Internal validation | 0.722 (0.694-0.750) | Good^†^ | 0.907 (0.890-0.925) | Good^†^ |
|  |  |  |  | External validation (Temporal validation) | 0.727 (0.670-0.784) | NI | 0.913 (0.878-0.947) | NI |
| 9 | 4 years | Logistic | Score chart | Internal validation only | 0.65 (0.62-0.68) | Good^*^ | 0.77 (0.74-0.79) | Good^*^ |
| 10 | 4 years | Logistic | Score chart | Internal validation only (random split-sample) | … | … | 0.799 (0.756-0.801) | NI |
| 11 | 6 years | Logistic | Score chart | Internal validation only | … | … | 0.714 (0.691-0.737) | Good^*^ |
| 12 | 3 years | Logistic | Score chart | Internal validation only (random split-sample) | 0.717 (0.703-0.731) | Good^†^ | 0.893 (0.883-0.902) | Good^†^ |
| 13 | 20 years | Competing | Score chart | Internal validation only | … | … | 0.76 (0.72-0.80) | Good^*^ |
| 14 | 5.4 years | Cox | Risk score (without corresponding absolute diabetes risk) | Internal validation only (random split-sample) | 0.67 | Good^‡^ | 0.77 | Good^‡^ |
| 15 | 6 years | Cox | Regression equation | Internal validation only (random split-sample) | … | … | 0.766 (0.742-0.789) | Good^*^ |
| 16 | 4.9 years | Cox | Regression equation | Internal validation only (random split-sample) | 0.70 (0.65-0.74) | Good^‡^ | 0.82 (0.78-0.85) | Good^‡^ |
| 17 | 4.2 years | Cox | Risk score (without corresponding absolute diabetes risk) | Internal validation only | … | … | 0.769 (0.739-0.798) | Good^*^ |

(continued)

| **Study No.** | **Prediction horizon** | **Modeling method** | **Presentation formats** | **Validation type** | **BASIC Model** | | **EXTENDED Model** | |
| --- | --- | --- | --- | --- | --- | --- | --- | --- |
|  |  |  |  |  | **C-Statistic** | **Calibration** | **C-Statistic** | **Calibration** |
| 18 | 6 years | Logistic | Risk score (without corresponding absolute diabetes risk) | Internal validation only (random split-sample) | 0.715 (0.672-0.757) | Good^*^ | … | … |
| 19 | 4.7 years | Logistic | Score chart | Internal validation only | … | … | 0.76 (0.73-0.79) | Good^*^ |
| 20 | 6 years | Cox | Regression equation | Internal validation only (random split-sample) | … | … | 0.791 (0.783-0.799) | Good^*^ |
| 21 | 10 years | Cox | Regression equation | Internal validation | … | … | 0.71 (0.70-0.73)^M^ 0.76 (0.75-0.78)^F^ | Good^‡^ |
|  |  |  |  | External validation (Geographical validation) | … | … | 0.63 (0.53-0.73)^M^  0.66 (0.55-0.76)^F^ | Good^‡^ |
| 22 | 5 years | Cox | Score chart | Internal validation only | … | … | 0.764 (0.750-0.777) | NI |
| 23 | 7 years | Cox | Score chart | Internal validation only (random split-sample) | 0.73 (0.72-0.74) | Good^‡^ | 0.89 (0.89-0.90) | Good^‡^ |
| 24 | 5 years | Cox | Score chart | Internal validation only | … | … | 0.735 | NI |
| 25 | 10 years | Cox | Score chart | Internal validation only | … | … | 0.77 | Good^*^ |
| 26 | 3 years | Cox | Nomogram | Internal validation only | … | … | 0.751 (0.729, 0.774)^M^ 0.863 (0.837, 0.888)^F^ | NI |
| 27 | 5 years | Cox | Nomogram | Internal validation only (random split-sample) | … | … | 0.862 (0.830-0.874) | Good^†^ |

(continued)

| **Study No.** | **Prediction horizon** | **Modeling method** | **Presentation formats** | **Validation type** | **BASIC Model** | | **EXTENDED Model** | |
| --- | --- | --- | --- | --- | --- | --- | --- | --- |
|  |  |  |  |  | **C-Statistic** | **Calibration** | **C-Statistic** | **Calibration** |
| 28 | 5 years | Cox | Regression equation (without reporting baseline hazard ratio) | Internal validation only | … | … | 0.75 (0.70-0.81) | NI |
| 29 | 5 years | Cox | Nomogram | Internal validation only (random split-sample) | … | … | 0.815 (0.797-0.834) | Good^‡^ |
| 30 | 7 years | Cox | Nomogram | Internal validation only | 0.73 (0.71-0.75) | Good^†^ | 0.74 (0.71 0.76) | Good^†^ |
| 31 | 3 and 5 years | Cox | Nomogram | Internal validation only (random split-sample) | … | … | 0.859 (0.850–0.868)^M^ 0.862 (0.853–0.871)^F^ | Good^‡^ |
| 32 | 5 years | Cox | Nomogram | Internal validation only (random split-sample) | … | … | 0.867 (0.840-0.894)^M^ 0.856 (0.795-0.917)^F^ | Good^‡^ |
| 33 | 10 years | Logistic | Nomogram | Internal validation only (random split-sample) | 0.804 (0.776-0.831) | NI | 0.904 (0.877-0.931) | NI |
| 34 | 7.75 years? | Cox | Nomogram | Internal validation only (random split-sample) | … | … | 0.87 (0.86-0.90) | NI |
| 35 | 3 years | Logistic | Nomogram | Internal validation (random split-sample) | … | … | 0.913 (0.889-0.936) | Underestimate |
| 36 | 8 years | Cox | Nomogram | Internal validation only (random split-sample) | … | … | 0.839 (0.804-0.874) | Good^†^ |

(continued)

| **Study No.** | **Prediction horizon** | **Modeling method** | **Presentation formats** | **Validation type** | **BASIC Model** | | **EXTENDED Model** | |
| --- | --- | --- | --- | --- | --- | --- | --- | --- |
|  |  |  |  |  | **C-Statistic** | **Calibration** | **C-Statistic** | **Calibration** |
| 37 | 5 years | Cox | Nomogram | Internal validation only (random split-sample) | … | … | 0.916 (0.889-0.943) | Good^†^ |
| 38 | 10 and 15 years | Cox | Nomogram | Internal validation only | … | … | 0.812 (0.729-0.895) | Good^‡^ |
| 39 | 3 years | Logistic | Nomogram | Internal validation only | … | … | 0.732 (0.688–0.776) | NI |
| 40 | 3 years | Logistic | Nomogram | Internal validation (random split-sample) | … | … | 0.933 | Good^†^ |
|  |  |  |  | External validation | … | … | 0.83 | Good^†^ |
| 41 | 5 years | Logistic | Risk score and regression equation | Internal validation | 0.610 (0.583-0.637) | Good^†^ | 0.757 (0.735-0.780) | Good^†^ |
|  |  |  |  | External validation | … | … | 0.643 (0.602-0.685) | Not good |
| 42 | 5 years | Logistic | Regression equation | Internal validation | 0.78 (0.76–0.81) | NI | … | … |
|  |  |  |  | External validation-1 | 0.66 (0.60–0.71) | Good^*^ | … | … |
|  |  |  |  | External validation-2  (Geographical validation) | 0.79 (0.72–0.86) | Good^*^ | … | … |
| 43 | 5 years | Cox | Regression equation | Internal validation only | … | … | 0.70 | NI |
| 44 | 10 years | Cox | Regression equation | Internal validation only (random split-sample) | 0.83 (0.83 to 0.84)^F^  0.81 (0.81 to 0.82)^M^ | Good^‡^ | 0.89 (0.88 to 0.89)^F^  0.87 (0.86 to 0.87)^M^ | Good^‡^ |

(continued)

| **Study No.** | **Prediction horizon** | **Modeling method** | **Presentation formats** | **Validation type** | **BASIC Model** | | **EXTENDED Model** | |
| --- | --- | --- | --- | --- | --- | --- | --- | --- |
|  |  |  |  |  | **C-Statistic** | **Calibration** | **C-Statistic** | **Calibration** |
| 45 | 7.5 years | Logistic | Regression equation | Internal validation only | … | … | 0.843 (0.818–0.867) | NI |
| 46 | 10 years | Logistic | Score chart | Internal validation | 0.86 | NI | … | … |
|  |  |  |  | External validation (Temporal validation) | 0.87 | NI | … | … |
| 47 | 9 years | Logistic | Regression equation | Internal validation only (random split-sample) | 0.71 (0.69-0.73) | NI | 0.80 (0.78-0.82) | NI |
| 48 | 7 years | Logistic | Score chart | Internal validation only | 0.724 | NI | 0.85 | NI |
| 49 | 2.5 years | Cox | Risk score and regression equation | Internal validation | … | … | 0.64 | NI |
|  |  |  |  | External validation-1 (Geographical validation) | … | … | 0.84 | NI |
|  |  |  |  | External validation-2 (Geographical validation) | … | … | 0.9 | NI |

^*^: The Hosmer-Lemeshow test showed good calibration. ^†^: The calibration plot and Hosmer-Lemeshow test showed good calibration. ^‡^: The calibration plot showed good calibration. ^M:^ Male only. ^F:^ Female only. NI, No information.

**Table S4** Predictors included in the BASIC models (N=21).

| **Study No.**  **Predictors** | **1** | **3** | **4** | **5** | **6** | **7** | **8** | **9** | **12** | **14** | **16** | **18** | **23** | **30** | **33** | **41** | **42** | **44** | **46** | **47** | **48** |
| --- | --- | --- | --- | --- | --- | --- | --- | --- | --- | --- | --- | --- | --- | --- | --- | --- | --- | --- | --- | --- | --- |
| Age | ● | … | ● | ● | ● | ● | ● | ● | ● | ● | ● | ● | ● | ● | ● | ● | ● | ● | ● | ● | ● |
| Sex | ● | ● | … | … | … | ● | ● | … | ● | ● | ● | … | ● | … | ● | … | ● | … | … | … | ● |
| Smoking | … | … | ● | … | ● | ● | ● | ● | ● | … | … | … | ● | … | ● | … | ● | ● | … | … | … |
| Family history | ● | ● | ● | ● | … | ● | ● | ● | … | ● | ● | ● | … | ● | … | … | ● | ● | … | ● | ● |
| Blood pressure* | ● | … | ● | ● | ● | ● | … | ● | ● | ● | ● | … | ● | … | ● | … | ● | ● | ● | ● | … |
| BMI | ● | ● | ● | ● | ● | ● | ● | ● | ● | ● | ● | ● | ● | ● | ● | ● | … | ● | ● | … | ● |
| Waist | ● | ● | ● | ● | … | ● | … | … | ● | … | … | ● | ● | … | ● | … | ● | … | ● | ● | … |
| Education | … | … | ● | ● | … | … | … | … | … | ● | … | … | … | ● | … | … | … | … | … | … | … |
| Physical inactivity | … | … | ● | … | … | ● | … | … | … | … | … | … | … | … | ● | … | ● | … | ● | … | … |
| High blood glucose | … | … | … | … | ● | … | … | … | … | … | … | … | … | … | … | … | ● | … | ● | … | … |
| Other non-invasive predictors | … | … | … | Alcohol | … | … | … | … | … | HR | … | … | … | WHR,  Sleep duration | Alcohol and others^†^ | Corticosteroids, Thiazide diuretic | … | Steroids and others^‡^ | Diet | Height, Ethnicity | … |
| **Numbers** | **6** | **4** | **8** | **7** | **4** | **8** | **5** | **5** | **6** | **7** | **5** | **4** | **6** | **6** | **17** | **4** | **8** | **15** | **7** | **6** | **4** |

^*^: Systolic blood pressure or hypertension history or use of antihypertensive drugs. ^†^: Nationality, alcohol, education, soft drink, tea, kcal, carbon, fat, protein, triceps, and sleep. ^‡^: Deprivation, atypical antipsychotics, statins, schizophrenia, or bipolar affective disorder, learning disability, gestational diabetes, and polycystic ovary syndrome in women. HR: Heart rate. WHR: Waist to hip. BMI: Body mass index.

**Table S5** Predictors included in the EXTENDED models (N=46).

| **Study No.**  **Predictors** | **1** | **2** | **3** | **4** | **5** | **6** | **7** | **8** | **9** | **10** | **11** | **12** | **13** | **14** | **15** | **16** | **18** | **19** | **20** | **21** | **22** |
| --- | --- | --- | --- | --- | --- | --- | --- | --- | --- | --- | --- | --- | --- | --- | --- | --- | --- | --- | --- | --- | --- |
| Age | ● | ● | … | ● | ● | ● | ● | ● | ● | … | … | ● | ● | ● | ● | ● | ● | ● |  | ● |  |
| Sex | ● | … | ● | ● | … | … | ● | ● | … | … | ● | ● | … | ● |  | ● | … | … | … | … | … |
| Smoking | … | … | … | ● | … | … | ● | ● | ● | … | … | ● | … | … | … | ● |  |  |  | ● | ● |
| Family history | ● | … | ● | ● | ● | … | ● | ● | ● | ● | … | … | … | ● | … | ● | ● | ● |  | ● | ● |
| Blood pressure | ● | … | … | ● | ● | ● | ● | … | ● | ● | ● | ● | … | ● | … | ● | ● | ● |  | ● | ● |
| BMI | ● | ● | ● | ● | ● | ● | ● | ● | ● | ● | ● | ● | ● | ● | ● |  | ● | ● | ● | ● | ● |
| Waist | ● |  | ● | ● | ● | … | ● | … | … | … | … | ● | … | … | … | … | … | … | … | … | … |
| Education | … | … | … | ● | ● | … |  | … | … | … | … | … | … | ● | … | … | … | … | … | … | … |
| Physical inactivity | … | … | … | ● | … | … | ● | … | … | … | … | … | ● | … | … | … | … | … | … | ● | … |
| FPG | … | ● | ● | ● | ● | ● | ● | ● | ● | ● | ● | ● | ● | ● | ● | ● | ● | ● | ● | ● | ● |
| 2hPG | ● | … | … | … | … | … | … | … | … | … | … | … | … | … | … | ● | … | … | … | … | … |
| HbA1c | … | … | … | … | … | … | … | ● | ● | … | ● | ● | … | … | … | ● |  | ● | … | … |  |
| TG | ● | ● | … | … | ● | ● | … | … | ● | ● | … | … | … | ● | ● | … | … | … | ● | … | ● |
| HDL-c | ● | ● | … | … | … | … | … | … | ● | ● | … | … | … | … | … | … | … | … | … | … | … |
| Other predictors | … | WBC | … | … | Alcohol | LDL-c, high blood glucose | … | … | … | … | CRP | … | Self-related health | HR | … | … | Diet | ALT | Tea,  WHTR | GGT, TC, Stain, alcohol | TC |
| Numbers | 9 | 6 | 5 | 10 | 9 | 7 | 9 | 7 | 9 | 6 | 6 | 8 | 5 | 9 | 4 | 8 | 6 | 7 | 5 | 12 | 7 |

(Continued)

(continued)

| **Study No.**  **Predictors** | **23** | **24** | **25** | **26** | **27** | **28** | **29** | **30** | **31** | **32** | **33** | **34** | **35** | **36** | **37** | **38** | **39** |
| --- | --- | --- | --- | --- | --- | --- | --- | --- | --- | --- | --- | --- | --- | --- | --- | --- | --- |
| Age | ● | ● | ● | ● | ● | ● | ● | ● | ● | ● | ● | ● | ● | … | ● | … | … |
| Sex | … | … | … | … | … | ● | ● | … | … | … | … | … | … | … | … | … | … |
| Smoking | ● | … | ● | … | … | ● | ● | … | … | ● | … | ● | … | ● | … | … | … |
| Family history | … | ● | ● | … | … | … | ● | ● | ● | … | … | … | … | … | … | ● | … |
| Blood pressure | ● | … | … | … | … | … | ● | … | … | ● | ● | … | ● | … | … | … | … |
| BMI | ● | ● | ● | ● | ● | ● | ● | ● | ● | ● | … | … | ● | … | … | … |  |
| Waist | … | … | … | … | … | … | … | … | … | … | ● | ● | … | ● |  | ● | … |
| Education | … | … | … | … | … | … | … | ● | … | … | … | … | … | … | … | … | … |
| Physical inactivity | … | … | … | … | … | ● | … | … | … | … | … | … | … | … | … | … | … |
| FPG | ● | … | ● | ● | ● | ● | … | ● | ● | ● | ● | ● | ● | ● | ● | ● | ● |
| 2hPG | … | … | … | … | … | … | … | … | … | … | … | … | … | … | … | … | ● |
| HbA1c | ● | … | … | … | … | … | … | … | … | ● | ● | ● | … | ● | ● | … | ● |
| TG | … | … | ● | ● | … | ● | … | … | ● | … | ● | … | ● | … | ● | ● | … |
| HDL-c | … | … | … | ● | … | … | … | … | … | … | … | … | … | … | … | … | … |
| Other predictors | dyslipidemia | TC | … | LDL-c | TC | … | Dyslipidemia | WHR, sleep duration | TC | Dyslipidemia | Alcohol and others^†^ | Fatty liver | … | … | fatty liver, GGT | Height, WBC | … |
| Numbers | 7 | 4 | 6 | 6 | 4 | 7 | 7 | 7 | 6 | 7 | 17 | 6 | 5 | 4 | 6 | 6 | 3 |

(continued)

| **Study No.**  **Predictors** | **40** | **41** | **43** | **44** | **45** | **47** | **48** | **49** |
| --- | --- | --- | --- | --- | --- | --- | --- | --- |
| Age | ● | ● | ● | … | ● | ● | ● | … |
| Sex | … | ● | ● | … | ● | … | ● | ● |
| Smoking | … | … | … | ● | … | … | … | … |
| Family history | … | … | ● | ● | ● | ● | ● | … |
| Blood pressure* | … | … | ● | ● | ● | ● | ● | ● |
| BMI | ● | ● | ● | ● | ● | … | ● | … |
| Waist | … | … | … | … | … | ● | ● | ● |
| Education | … | … | … | … | … | … | … | … |
| Physical activity | … | … | … | … | … | … | … | … |
| FPG | ● | ● | ● | ● | ● | ● | ● | ● |
| 2hPG | … | ● | ● |  | … | … | … | … |
| HbA1c | … | ● | ● | ● | … | … | … | … |
| TG | … | … | … | … | … | ● | ● | ● |
| HDL-c | ● | … | ● | … | ● | ● | ● | … |
| Other predictors | LDL-c,  ALT | Corticosteroids, Thiazide diuretic | LDL-c, CVD, Ethnicity | Steroids, others^‡^ | Ethnicity | Ethnicity, height | … | CVD, acarbose, height |
| Numbers | 6 | 8 | 12 | 16 | 8 | 9 | 9 | 8 |

^*^: Systolic blood pressure or hypertension history or use of antihypertensive drugs. ^†^: Nationality, alcohol, education, soft drink, tea, kcal, carbon, fat, protein, triceps, and sleep. ^‡^: Deprivation, atypical antipsychotics, statins, schizophrenia, or bipolar affective disorder, learning disability, gestational diabetes, and polycystic ovary syndrome in women. BMI: Body mass index. GGT: Gamma-glutamyl transferase. TC: Total cholesterol. LDL-c: Low density cholesterol. HDL-c: High density cholesterol. CVD: Cardiovascular diseases. HR: Heart rate. WBC: White blood cell. WHR: Waist to hip ratio.

**Table S6** Baseline characteristics of the participants with intermediate hyperglycemia at baseline of the ACE, Luzhou, and TCLSIH cohorts.

| **Variables** | **ACE cohort** | **Luzhou cohort** | **TCLSIH cohort** |
| --- | --- | --- | --- |
| Numbers | 3241 | 1333 | 1702 |
| Age (years) | 63.0 (57.0, 70.0) | 60.0 (54.0, 66.0) | 53.5 (47.2, 61.0) |
| Sex (Male) | 2357 (72.7%) | 459 (34.4%) | 1281 (75.3%) |
| Smoking status |  |  |  |
| Never Smoker | 1422 (43.9%) | 1108 (83.1%) | 1034 (60.8%) |
| Ex-smoker | 1346 (41.5%) | 71 (5.3%) | 140 (8.2%) |
| Current smoker | 473 (14.6%) | 154 (11.6%) | 528 (31.0%) |
| Currently taking alcohol | 380 (11.7%) | 124 (9.3%) | 1037 (60.9%) |
| Weight (kg) | 70.0 (63.0, 78.0) | 60.0 (54.0, 66.5) | 76.2 (68.2, 83.8) |
| Height (cm) | 167 (160, 171) | 157 (152, 163) | 170 (163, 174) |
| Waist circumference (cm) | 91.0 (86.0, 97.0) | 84.3 (78.1, 91.0) | 92.0 (85.0, 97.8) |
| Waist to height ratio (cm/cm) | 0.55 (0.52, 0.59) | 0.54 (0.50, 0.58) | 0.54 (0.51, 0.57) |
| Body mass index (kg/m^2^) | 25.4 (23.5, 27.6) | 24.4 (22.3, 26.5) | 26.7 (24.5, 28.8) |
| Systolic blood pressure (mmHg) | 130 (120, 140) | 127 (114, 141) | 130 (120, 140) |
| Diastolic blood pressure (mmHg) | 80.0 (70.0, 85.0) | 77.3 (70.7, 85.0) | 80.0 (75.0, 90.0) |
| Resting heart Rate | NA | 79.3 (73.3, 87.0) | NA |
| Prior hypertension history | 2109 (65.1%) | 297 (22.3%) | 579 (34.0%) |
| Prior cardiovascular disease | 3241 (100%) | 75 (5.6%) | 167 (9.8%) |
| Family history of diabetes | NA | 128 (9.6%) | 453 (26.6%) |
| Physical inactivity | NA | 379 (28.4%) | NA |
| Education Level (high school or more) | NA | 377 (28.3%) | NA |
| Drinking tea frequently | NA | 274 (20.6%) | NA |
| Statin | 2969 (91.6%) | 1 (0.1%) | NA |
| Steroid | 35 (1.1%) | 0 (0.0%) | NA |
| Self-related health | 85.0 (78.0, 90.0) | NA | NA |
| Fasting plasma glucose (mmol/L) | 5.41 (5.00, 5.90) | 5.55 (5.21, 6.00) | 5.60 (5.00, 6.20) |
| 2-hour plasma glucose (mmol/L) | 9.10 (8.35, 10.1) | 8.78 (8.15, 9.67) | 8.30 (7.90, 9.20) |
| HbA1c (%) | 5.90 (5.60, 6.30) | 6.00 (5.70, 6.20) | 5.70 (5.40, 6.10) |
| Triglycerides (mmol/L) | 1.43 (1.04, 1.96) | 1.40 (0.97, 2.06) | 1.76 (1.24, 2.53) |
| HDL-Cholesterol (mmol/L) | 1.12 (0.96, 1.33) | 1.18 (0.98, 1.41) | 1.21 (1.03, 1.45) |
| LDL-Cholesterol (mmol/L) | 2.23 (1.77, 2.85) | 2.56 (2.04, 3.11) | 3.15 (2.62, 3.68) |
| Total cholesterol (mmol/L) | 4.05 (3.47, 4.78) | 4.62 (3.84, 5.34) | 5.25 (4.68, 5.90) |
| White blood cell (*10^9/L) | 6.12 (5.20, 7.30) | NA | 5.81 (5.00, 6.82) |
| Alanine aminotransferase (U/L) | 22.0 (16.4, 31.0) | 13.0 (9.00, 19.0) | 24.0 (18.0, 36.0) |
| Follow-up duration (years) | 5.0 | 3.0 | 3.0 |
| Diabetes progression during follow-up | 509 (15.7%) | 260(19.5%) | 396(23.3%) |

**Table S7** The validation results of the included BASIC models in intermediate hyperglycemia participants of the ACE, Luzhou, and TCLSIH cohorts.

| **Study**  **No.** | **ACE cohort** | | | **Luzhou cohort** | | | **TCLSIH cohort** | | |
| --- | --- | --- | --- | --- | --- | --- | --- | --- | --- |
|  | **C-statistic**  **(95% CI)** | **Original**  **P/0 (95% CI)** | **Recalibrated P/0 (95% CI)** | **C-statistic**  **(95% CI)** | **Original**  **P/0 (95% CI)** | **Recalibrated P/0 (95%CI)** | **C-statistic**  **(95% CI)** | **Original**  **P/0 (95%CI)** | **Recalibrated P/0 (95% CI)** |
| 1 | 0.58(0.55-0.6) | NA | 1.34(1.24-1.45) | 0.57(0.53-0.60) | NA | 1.34(1.20-1.50) | 0.64(0.61-0.67) | NA | 1.41(1.29-1.54) |
| 3 | 0.59(0.56-0.61) | 1.08(1.00-1.17) | 1.15(1.06-1.25) | 0.54(0.50-0.58) | 0.35(0.31-0.39) | 1.16(1.04-1.30) | 0.59(0.56-0.62) | 0.44(0.40-0.48) | 1.14(1.04-1.24) |
| 4 | 0.57(0.54-0.59) | NA | 1.25(1.15-1.35) | 0.58(0.54-0.62) | NA | 1.32(1.18-1.48) | 0.62(0.59-0.65) | NA | 1.32(1.21-1.44) |
| 5 | 0.57 (0.54-0.59) | 0.39(0.36-0.42) | 1.00(0.92-1.09) | 0.58(0.54-0.62) | 0.20(0.18-0.23) | 1.00(0.90-1.12) | 0.62(0.59-0.65) | 0.21(0.19-0.23) | 1.00(0.92-1.09) |
| 6 | 0.52(0.50-0.55) | NA | 1.54(1.42-1.67) | 0.58(0.54-0.62) | NA | 1.32(1.18-1.47) | 0.55(0.52-0.58) | NA | 1.27(1.17-1.39) |
| 7 | 0.6(0.57-0.62) | 0.39(0.36-0.42) | 1.20(1.10-1.30) | 0.55(0.51-0.59) | 0.16(0.14-0.18) | 1.17(1.05-1.31) | 0.62(0.59-0.65) | 0.18(0.17-0.2) | 1.20(1.11-1.32) |
| 8 | 0.59(0.57-0.62) | 0.52(0.48-0.56) | 0.99(0.91-1.07) | 0.54(0.50-0.57) | 0.11(0.1-0.12) | 0.93(0.84-1.04) | 0.61(0.57-0.64) | 0.18(0.17-0.2) | 0.94(0.86-1.03) |
| 9 | 0.57(0.54-0.59) | NA | 1.16(1.07-1.26) | 0.58(0.54-0.62) | NA | 1.17(1.05-1.31) | 0.63(0.60-0.66) | NA | 1.20(1.10-1.31) |
| 12 | 0.59(0.56-0.61) | 1.07(0.98-1.16) | 1.28(1.18-1.39) | 0.56(0.52-0.60) | 0.32(0.29-0.36) | 1.36(1.21-1.52) | 0.59(0.56-0.62) | 0.41(0.38-0.45) | 1.39(1.27-1.52) |
| 13 | 0.58(0.56-0.61) | NA | 1.11(1.02-1.20) | 0.54(0.51-0.58) | NA | 1.13(1.01-1.26) | 0.61(0.58-0.64) | NA | 1.12(1.03-1.22) |
| 16 | 0.53(0.50-0.55) | 1.37(1.27-1.49) | 1.18(1.09-1.28) | 0.56(0.52-0.60) | 0.30(0.27-0.33) | 1.19(1.06-1.33) | 0.61(0.58-0.64) | 0.31(0.29-0.34) | 1.16(1.06-1.26) |
| 18 | 0.56(0.53-0.58) | NA | 1.27(1.17-1.38) | 0.57(0.54-0.61) | NA | 1.42(1.28-1.59) | 0.63(0.60-0.66) | NA | 1.43(1.31-1.56) |
| 23 | 0.60(0.57-0.62) | 0.92(0.85-1.00) | 1.24(1.15-1.35) | 0.56(0.52-0.60) | 0.26(0.23-0.29) | 1.25(1.12-1.40) | 0.58(0.55-0.62) | 0.36(0.33-0.39) | 1.29(1.18-1.41) |
| 30 | 0.54(0.51-0.57) | 2.65(2.45-2.88) | 1.04(0.96-1.13) | 0.58(0.54-0.61) | 0.48(0.43-0.53) | 1.12(1.00-1.25) | 0.63(0.60-0.66) | 0.43(0.39-0.47) | 1.09(1.00-1.19) |
| 33 | 0.57(0.54-0.59) | 0.79(0.73-0.86) | 1.00(0.93-1.09) | 0.55(0.51-0.59) | 0.34(0.30-0.38) | **1.01(0.90-1.12)**^*^ | 0.56(0.53-0.59) | 0.36(0.33-0.39) | **1.00(0.92-1.10)**^*^ |
| 41 | NA^†^ | NA^†^ | NA^†^ | 0.50(0.46-0.54) | 1.17(1.05-1.31) | NA | 0.50(0.46-0.53) | 1.00(0.92-1.09) | **1.00(0.91-1.09)**^*^ |
| 42 | 0.56(0.53-0.58) | 1.40(1.29-1.52) | 1.02(0.94-1.11) | 0.57(0.53-0.61) | 0.33(0.29-0.36) | 0.96(0.86-1.07) | 0.63(0.59-0.66) | 0.37(0.34-0.41) | 0.97(0.89-1.06) |
| 44 | 0.55(0.52-0.57) | 1.77(1.63-1.92) | 1.65(1.52-1.79) | 0.57(0.53-0.61) | 0.14(0.12-0.15) | 1.25(1.12-1.4) | 0.63(0.60-0.66) | 0.30(0.27-0.32) | 1.28(1.18-1.40) |
| 46 | 0.56(0.54-0.59) | 0.11(0.11-0.12) | 0.93(0.86-1.01) | 0.56(0.52-0.6) | 0.03(0.03-0.04) | 0.92(0.83-1.03) | 0.58(0.55-0.61) | 0.04(0.04-0.04) | 0.87(0.79-0.95) |
| 47 | 0.54(0.51-0.56) | 1.09(1-1.18) | 1.04(0.96-1.12) | 0.59(0.55-0.63) | 0.27(0.25-0.31) | 0.98(0.88-1.10) | 0.61(0.58-0.64) | 0.26(0.23-0.28) | 0.98(0.90-1.07) |
| 48 | 0.58(0.56-0.61) | 0.58(0.54-0.63) | 0.99(0.91-1.07) | 0.56(0.52-0.6) | 0.13(0.12-0.15) | 0.95(0.85-1.06) | 0.60(0.56-0.63) | 0.17(0.16-0.19) | 0.95(0.87-1.04) |

^*^: These models showed *P*<0.05 in the Hosmer-Lemeshow test which indicated goodness of fit. ^†:^ The validation in ACE cohort was not conducted for this study because it was developed based on the ACE cohort. NA: not applicable.

**Table S8** The validation results of the included EXTENDED models in intermediate hyperglycemia participants of the ACE, Luzhou, and TCLSIH cohorts.

| **Study**  **No.** | **ACE cohort** | | | **Luzhou cohort** | | | **TCLSIH cohort** | | | |
| --- | --- | --- | --- | --- | --- | --- | --- | --- | --- | --- |
|  | **C-statistic**  **(95% CI)** | **Original**  **P/0 (95% CI)** | **Recalibrated P/0 (95% CI)** | **C-statistic**  **(95% CI)** | **Original**  **P/0 (95% CI)** | **Recalibrated P/0 (95% CI)** | **C-statistic**  **(95% CI)** | **Original**  **P/0 (95% CI)** | **Recalibrated P/0 (95% CI)** |  |
| 1 | 0.60(0.57-0.62) | NA | 1.35(1.24-1.46) | 0.55(0.52-0.59) | NA | 1.49(1.33-1.66) | 0.61(0.57-0.64) | NA | 1.62(1.48-1.77) |  |
| 2 | 0.68(0.66-0.71) | 1.16(1.07-1.26) | 1.29(1.19-1.4) | 0.61(0.57-0.64) | 0.57(0.51-0.64) | 1.23(1.10-1.38) | 0.70(0.67-0.72) | 0.55(0.50-0.60) | 1.31(1.20-1.43) |  |
| 3 | 0.68(0.66-0.71) | NA | 1.42(1.31-1.54) | 0.59(0.55-0.63) | NA | 1.39(1.24-1.55) | 0.71(0.69-0.74) | NA | 1.40(1.28-1.53) |  |
| 4 | 0.69(0.66-0.71) | 1.12(1.03-1.21) | 2.38(2.20-2.59) | 0.62(0.58-0.66) | 0.31(0.27-0.34) | 2.09(1.87-2.34) | 0.74(0.71-0.77) | 0.43(0.40-0.47) | 2.23(2.04-2.43) |  |
| 5 | 0.68 (0.65-0.70) | 0.67(0.62-0.73) | 1.00(0.92-1.09) | 0.62(0.58-0.66) | 0.36(0.32-0.40) | 1.00(0.90-1.12) | 0.72(0.69-0.75) | 0.37(0.34-0.4) | 1.00(0.92-1.09) |  |
| 6 | 0.64(0.61-0.66) | NA | 1.24(1.14-1.34) | 0.6(0.56-0.64) | NA | 1.23(1.1-1.38) | 0.68(0.65-0.71) | NA | 1.16(1.07-1.27) |  |
| 7 | 0.68(0.66-0.71) | 0.41(0.37-0.44) | 1.50(1.38-1.62) | 0.59(0.55-0.63) | 0.12(0.11-0.13) | 1.37(1.23-1.54) | 0.72(0.70-0.75) | 0.16(0.14-0.17) | 1.48(1.36-1.62) |  |
| 8 | 0.69(0.66-0.71) | 0.75(0.69-0.81) | 0.99(0.91-1.07) | 0.59(0.55-0.62) | 0.32(0.29-0.36) | 0.93(0.83-1.04) | 0.72(0.69-0.75) | 0.34(0.31-0.37) | 0.90(0.83-0.99) |  |
| 9 | 0.69(0.66-0.71) | 2.78(2.57-3.01) | 1.77(1.63-1.92) | 0.62(0.58-0.65) | 1.19(1.07-1.33) | 1.57(1.4-1.75) | 0.73(0.70-0.75) | 1.07(0.98-1.17) | 1.73(1.58-1.89) |  |
| 10 | 0.67(0.65-0.7) | 0.90(0.83-0.98) | 5.76(5.32-6.25) | 0.60(0.57-0.64) | 0.28(0.25-0.31) | 4.69(4.21-5.25) | 0.71(0.69-0.74) | 0.32(0.29-0.34) | 3.99(3.66-4.36) |  |
| 11 | 0.70(0.68-0.72) | 3.40(3.13-3.68) | 1.35(1.24-1.46) | 0.63(0.59-0.67) | 1.57(1.41-1.76) | 1.29(1.16-1.45) | 0.72(0.69-0.75) | 1.28(1.17-1.39) | 1.38(1.27-1.51) |  |
| 12 | 0.71(0.68-0.73) | 1.39(1.28-1.51) | 2.42(2.23-2.63) | 0.62(0.59-0.66) | 0.52(0.47-0.58) | 2.21(1.98-2.47) | 0.74(0.71-0.76) | 0.65(0.60-0.71) | 2.07(1.90-2.26) |  |
| 13 | 0.60(0.58-0.63) | 0.17(0.16-0.18) | 1.31(1.21-1.42) | 0.52(0.48-0.56) | 0.08(0.07-0.08) | 1.24(1.11-1.39) | 0.59(0.56-0.62) | 0.08(0.07-0.08) | 1.19(1.09-1.30) |  |
| 14 | 0.68(0.66-0.71) | NA | 1.46(1.35-1.58) | 0.60(0.57-0.64) | NA | 1.43(1.28-1.6) | 0.73(0.70-0.75) | NA | 1.46(1.34-1.60) |  |
| 15 | 0.67(0.65-0.69) | 0.27(0.25-0.3) | 1.38(1.28-1.5) | 0.61(0.57-0.64) | 0.14(0.12-0.15) | 1.37(1.23-1.54) | 0.70(0.67-0.73) | 0.13(0.12-0.14) | 1.42(1.3-1.55) |  |
| 16 | 0.72(0.70-0.74) | 3.57(3.29-3.87) | 1.83(1.69-1.99) | 0.66(0.62-0.69) | 1.24(1.11-1.39) | 1.58(1.42-1.77) | 0.74(0.71-0.76) | 0.90(0.83-0.99) | 1.52(1.39-1.66) |  |
| 17 | 0.66(0.64-0.69) | NA | 1.51(1.4-1.64) | 0.61(0.57-0.65) | NA | 1.45(1.30-1.62) | 0.70(0.67-0.73) | NA | 1.69(1.55-1.85) |  |
| 19 | 0.69(0.67-0.72) | 0.82(0.75-0.88) | 1.52(1.41-1.65) | 0.62(0.59-0.66) | 0.48(0.43-0.53) | 1.55(1.39-1.74) | 0.66(0.63-0.69) | 0.41(0.37-0.44) | 1.49(1.36-1.62) |  |
| 20 | 0.67(0.65-0.7) | 0.29(0.27-0.32) | 1.56(1.44-1.69) | 0.60(0.56-0.64) | 0.15(0.13-0.16) | 1.52(1.37-1.71) | 0.70(0.67-0.73) | 0.17(0.16-0.18) | 1.56(1.43-1.71) |  |
| 21 | 0.63(0.61-0.66) | 1.33(1.23-1.44) | 1.73(1.6-1.88) | 0.62(0.58-0.65) | 0.23(0.21-0.26) | 1.34(1.20-1.50) | 0.72(0.69-0.74) | 0.34(0.31-0.37) | 1.38(1.27-1.51) |  |
| 22 | 0.69(0.66-0.71) | 2.25(2.07-2.44) | 1.41(1.3-1.53) | 0.60(0.56-0.64) | 0.95(0.85-1.06) | 1.33(1.19-1.48) | 0.73(0.7-0.75) | 0.93(0.86-1.02) | 1.39(1.28-1.52) |  |
| 23 | 0.71(0.69-0.74) | 0.74(0.68-0.8) | 2.09(1.93-2.27) | 0.62(0.59-0.66) | 0.34(0.3-0.38) | 1.94(1.74-2.17) | 0.74(0.71-0.76) | 0.48(0.44-0.52) | 1.94(1.78-2.11) |  |
| 24 | 0.56(0.54-0.59) | 5.45(5.03-5.91) | 1.34(1.24-1.46) | 0.53(0.49-0.57) | 2.10(1.88-2.35) | 1.40(1.25-1.56) | 0.62(0.59-0.65) | 1.88(1.73-2.06) | 1.35(1.24-1.47) |  |
| 25 | 0.69(0.66-0.71) | 0.72(0.66-0.78) | 0.92(0.85-1.00) | 0.61(0.57-0.65) | 0.33(0.29-0.37) | 0.88(0.79-0.99) | 0.73(0.7-0.75) | 0.47(0.43-0.51) | 0.88(0.81-0.96) |  |
| 26 | 0.60(0.57-0.62) | 4.74(4.37-5.14) | 1.01(0.94-1.1) | 0.60(0.56-0.64) | 2.30(2.06-2.57) | **1.02(0.91-1.14)**^*^ | 0.64(0.61-0.67) | 2.19(2.01-2.39) | 1.01(0.93-1.11) |  |
| 27 | 0.67(0.64-0.69) | 1.83(1.69-1.99) | 1.01(0.93-1.1) | 0.62(0.58-0.66) | 0.92(0.82-1.03) | 1.01(0.9-1.13) | 0.71(0.68-0.73) | 0.88(0.81-0.97) | 1.01(0.93-1.1) |  |
| 28 | 0.67(0.64-0.70) | NA | 1.34(1.24-1.46) | 0.59(0.55-0.63) | NA | 1.34(1.2-1.5) | 0.69(0.67-0.72) | NA | 1.34(1.23-1.46) |  |
| 29 | 0.54(0.52-0.57) | 2.53(2.34-2.75) | 1.30(1.2-1.41) | 0.56(0.52-0.6) | 0.46(0.41-0.51) | 1.36(1.22-1.52) | 0.62(0.59-0.65) | 0.54(0.5-0.59) | 1.39(1.27-1.51) |  |
| 30 | 0.64(0.61-0.66) | 2.52(2.33-2.73) | 1.15(1.06-1.25) | 0.59(0.55-0.63) | 0.70(0.63-0.78) | 1.14(1.02-1.28) | 0.73(0.7-0.76) | 0.63(0.58-0.69) | 1.13(1.03-1.23) |  |
| 31 | 0.65(0.63-0.68) | 2.56(2.37-2.78) | 1.02(0.94-1.11) | 0.61(0.57-0.65) | 1.14(1.02-1.28) | **1.02(0.91-1.14)**^*^ | 0.70(0.67-0.73) | 1.21(1.11-1.33) | 1.02(0.94-1.12) |  |
| 32 | 0.68(0.66-0.71) | 0.79(0.73-0.86) | 1.28(1.18-1.39) | 0.60(0.56-0.64) | 0.34(0.31-0.38) | 1.25(1.12-1.4) | 0.70(0.67-0.72) | 0.34(0.31-0.37) | 1.31(1.20-1.43) |  |
| 33 | 0.69(0.67-0.72) | 0.68(0.63-0.74) | 1.05(0.97-1.14) | 0.64(0.6-0.68) | 0.32(0.29-0.36) | 1.04(0.93-1.17) | 0.71(0.68-0.74) | 0.28(0.26-0.3) | 1.04(0.96-1.14) |  |
| 34 | 0.66(0.63-0.68) | 2.58(2.38-2.8) | 1.05(0.97-1.13) | 0.60(0.56-0.64) | 1.01(0.91-1.13) | **1.04(0.93-1.17)**^*^ | 0.70(0.67-0.73) | 0.78(0.71-0.85) | 1.05(0.96-1.14) |  |
| 35 | 0.67(0.65-0.7) | 1.69(1.56-1.84) | 1.01(0.93-1.1) | 0.62(0.58-0.65) | 0.84(0.75-0.94) | 1.01(0.9-1.13) | 0.72(0.69-0.75) | 0.80(0.73-0.87) | 1.01(0.93-1.11) |  |
| 36 | 0.70(0.68-0.73) | 2.01(1.86-2.18) | 1.04(0.96-1.13) | 0.63(0.59-0.67) | 0.93(0.83-1.04) | 1.03(0.92-1.15) | 0.73(0.7-0.76) | 0.77(0.7-0.84) | 1.04(0.96-1.14) |  |
| 37 | 0.69(0.67-0.72) | 4.82(4.45-5.23) | 1.03(0.95-1.12) | 0.64(0.6-0.68) | 1.85(1.66-2.08) | 1.02(0.91-1.14) | 0.74(0.71-0.77) | 1.36(1.25-1.48) | 1.03(0.94-1.12) |  |
| 38 | 0.62(0.59-0.65) | 1.44(1.33-1.56) | 1.00(0.92-1.08) | 0.59(0.55-0.63) | 0.44(0.39-0.49) | 1.00(0.9-1.12) | 0.65(0.62-0.68) | 0.49(0.45-0.54) | 1.00(0.92-1.09) |  |
| 39 | 0.72(0.69-0.74) | 2.27(2.10-2.46) | 1.05(0.97-1.14) | 0.65(0.61-0.69) | 1.16(1.04-1.3) | 1.03(0.93-1.16) | 0.75(0.73-0.78) | 0.78(0.72-0.86) | 1.05(0.96-1.15) |  |
| 40 | 0.66(0.63-0.69) | 2.94(2.71-3.19) | 1.01(0.94-1.10) | 0.61(0.57-0.65) | 1.42(1.27-1.59) | 1.01(0.91-1.13) | 0.71(0.68-0.74) | 1.31(1.2-1.43) | 1.01(0.93-1.11) |  |
| 41 | NA^†^ | NA^†^ | NA^†^ | 0.64(0.61-0.68) | 0.87(0.78-0.97) | 1.05(0.94-1.17) | 0.74(0.71-0.77) | 0.70(0.64-0.77) | 0.94(0.86-1.02) |  |
| 43 | **0.73(0.71-0.75)** | 1.73(1.59-1.87) | 1.33(1.22-1.44) | **0.67(0.63-0.70)** | 0.71(0.63-0.79) | 1.20(1.07-1.34) | **0.78(0.76-0.81)** | 0.48(0.44-0.52) | 1.19(1.10-1.3) |  |
| 44 | 0.67(0.65-0.7) | 1.80(1.66-1.95) | 2.16(1.99-2.34) | 0.62(0.58-0.66) | 0.26(0.24-0.29) | 1.58(1.42-1.77) | 0.74(0.72-0.77) | 0.43(0.4-0.48) | 1.74(1.59-1.90) |  |
| 45 | 0.67(0.64-0.69) | 1.78(1.64-1.93) | 1.25(1.15-1.36) | 0.62(0.58-0.66) | 0.70(0.62-0.78) | 1.09(0.98-1.22) | 0.72(0.69-0.75) | 0.53(0.49-0.58) | 1.04(0.96-1.14) |  |
| 47 | 0.68(0.65-0.70) | 1.17(1.08-1.27) | 1.17(1.08-1.27) | 0.62(0.58-0.66) | 0.44(0.39-0.49) | 1.03(0.92-1.15) | 0.73(0.70-0.76) | 0.37(0.34-0.41) | 0.99(0.91-1.09) |  |
| 48 | 0.69(0.66-0.71) | 1.94(1.79-2.1) | 1.51(1.40-1.64) | 0.62(0.58-0.66) | 0.82(0.74-0.92) | 1.26(1.13-1.41) | 0.72(0.70-0.75) | 0.81(0.74-0.88) | 1.34(1.23-1.47) |  |
| 49 | 0.67(0.64-0.69) | 4.91(4.53-5.33) | 1.07(0.98-1.16) | 0.62(0.58-0.66) | 1.47(1.32-1.64) | **1.05(0.94-1.17)**^*^ | 0.69(0.66-0.72) | 1.29(1.18-1.41) | 1.05(0.96-1.15) |  |

^*^: These models showed *P*<0.05 in the Hosmer-Lemeshow test which indicated goodness of fit. ^†:^ The validation in ACE cohort was not conducted for this study because it was developed based on the ACE cohort. CI: Confidence interval. NA: not applicable.

**Table S9** The validation results of the included BASIC models in non-diabetic participants of the Luzhou and TCLSIH cohorts.

| **Study**  **No.** | **Luzhou cohort** | | | **TCLSIH cohort** | | |
| --- | --- | --- | --- | --- | --- | --- |
|  | **C-statistic**  **(95% CI)** | **Original**  **P/0 (95% CI)** | **Recalibrated**  **P/0 (95% CI)** | **C-statistic**  **(95% CI)** | **Original**  **P/0 (95% CI)** | **Recalibrated**  **P/0 (95% CI)** |
| 1 | 0.62(0.59-0.65) | NA | 1.47(1.34-1.62) | 0.75(0.73-0.77) | NA | 2.00(1.85-2.17) |
| 3 | 0.59(0.56-0.62) | 0.57(0.52-0.63) | 1.23(1.12-1.36) | 0.70(0.68-0.72) | 1.60(1.48-1.73) | 1.34(1.24-1.46) |
| 4 | 0.64(0.61-0.67) | NA | 1.46(1.33-1.61) | 0.76(0.74-0.77) | NA | 1.80(1.66-1.95) |
| 5 | 0.64(0.61-0.67) | 0.29(0.26-0.32) | 1.00(0.91-1.1) | 0.74(0.72-0.76) | 0.59(0.54-0.64) | 1.00(0.92-1.09) |
| 6 | 0.62(0.59-0.65) | NA | 1.39(1.26-1.53) | 0.69(0.67-0.71) | NA | 1.51(1.39-1.63) |
| 7 | 0.60(0.57-0.63) | 0.25(0.23-0.28) | 1.23(1.12-1.35) | 0.73(0.71-0.75) | 0.57(0.52-0.62) | 1.46(1.35-1.58) |
| 8 | 0.58(0.55-0.61) | 0.17(0.16-0.19) | 0.96(0.87-1.05) | 0.71(0.69-0.73) | 0.58(0.54-0.63) | 1.00(0.92-1.08) |
| 9 | 0.63(0.6-0.66) | NA | 1.23(1.11-1.35) | 0.75(0.73-0.77) | NA | 1.43(1.32-1.55) |
| 12 | 0.63(0.6-0.66) | 0.48(0.43-0.53) | 1.52(1.38-1.67) | 0.74(0.72-0.76) | 1.22(1.13-1.32) | 2.10(1.93-2.27) |
| 13 | 0.60(0.57-0.63) | NA | 1.17(1.06-1.29) | 0.72(0.7-0.74) | NA | 1.29(1.19-1.39) |
| 16 | 0.63(0.6-0.65) | 0.43(0.39-0.47) | 1.27(1.16-1.4) | 0.76(0.74-0.78) | 0.85(0.79-0.93) | 1.37(1.27-1.49) |
| 18 | 0.63(0.6-0.66) | NA | 1.59(1.45-1.75) | 0.74(0.72-0.76) | NA | 2.09(1.93-2.27) |
| 23 | 0.62(0.59-0.65) | 0.39(0.36-0.43) | 1.35(1.23-1.49) | 0.73(0.71-0.75) | 1.06(0.97-1.14) | 1.73(1.6-1.88) |
| 30 | 0.62(0.59-0.64) | 0.78(0.71-0.86) | 1.17(1.07-1.29) | 0.71(0.69-0.73) | 0.58(0.54-0.63) | 1.17(1.08-1.27) |
| 33 | 0.62(0.59-0.65) | 0.58(0.53-0.64) | 1.01(0.92-1.11) | 0.70(0.68-0.72) | 1.35(1.25-1.47) | 1.01(0.93-1.10) |
| 41 | 0.51(0.48-0.54) | 2.06(1.87-2.27) | NA | 0.55(0.52-0.57) | 2.20(2.03-2.39) | 1.02(0.94-1.11) |
| 42 | 0.62(0.59-0.65) | 0.49(0.45-0.54) | 0.99(0.9-1.09) | 0.76(0.74-0.77) | 1.28(1.18-1.38) | 1.06(0.98-1.15) |
| 44 | 0.62(0.59-0.65) | 0.20(0.18-0.22) | 1.36(1.23-1.49) | 0.76(0.74-0.78) | 0.50(0.46-0.54) | 1.74(1.61-1.89) |
| 46 | 0.60(0.58-0.63) | 0.05(0.04-0.05) | 0.95(0.87-1.05) | 0.69(0.67-0.71) | 0.12(0.11-0.13) | 0.96(0.89-1.04) |
| 47 | 0.66(0.63-0.69) | 0.41(0.37-0.45) | 1.01(0.91-1.11) | 0.74(0.72-0.76) | 0.72(0.66-0.78) | 1.04(0.96-1.12) |
| 48 | 0.61(0.58-0.63) | 0.21(0.19-0.23) | 0.98(0.89-1.07) | 0.71(0.69-0.73) | 0.54(0.5-0.59) | 1.01(0.93-1.09) |

CI: Confidence interval. NA: not applicable.

**Table S10** The validation results of the included EXTENDED models in non-diabetic participants of the Luzhou and TCLSIH cohorts.

| **Study**  **No.** | **Luzhou cohort** | | | **TCLSIH cohort** | | |
| --- | --- | --- | --- | --- | --- | --- |
|  | **C-statistic**  **(95% CI)** | **Original**  **P/0 (95% CI)** | **Recalibrated P/0 (95% CI)** | **C-statistic**  **(95% CI)** | **Original**  **P/0 (95% CI)** | **Recalibrated P/0**  **(95% CI)** |
| 1 | 0.69(0.67-0.72) | NA | 1.92(1.75-2.12) | 0.82(0.81-0.84) | NA | 2.39(2.21-2.60) |
| 2 | 0.65(0.62-0.68) | 0.82(0.74-0.9) | 1.31(1.19-1.44) | 0.84(0.82-0.85) | 1.49(1.38-1.62) | 1.56(1.44-1.70) |
| 3 | 0.64(0.61-0.67) | NA | 1.45(1.32-1.6) | 0.83(0.81-0.85) | NA | 1.55(1.43-1.68) |
| 4 | 0.67(0.64-0.7) | 0.31(0.29-0.35) | 4.79(4.36-5.28) | 0.86(0.84-0.88) | 0.55(0.51-0.60) | 3.62(3.34-3.93) |
| 5 | 0.68(0.65-0.71) | 0.39(0.36-0.43) | 1.00(0.91-1.1) | 0.84(0.83-0.86) | 0.59(0.54-0.64) | 1(0.92-1.09) |
| 6 | 0.66(0.63-0.69) | NA | 1.29(1.17-1.42) | 0.82(0.8-0.84) | NA | 1.27(1.17-1.38) |
| 7 | 0.64(0.61-0.67) | 0.17(0.15-0.18) | 1.39(1.26-1.53) | 0.84(0.82-0.86) | 0.39(0.36-0.42) | 1.62(1.50-1.76) |
| 8 | 0.64(0.61-0.67) | 0.38(0.34-0.42) | 0.95(0.86-1.04) | 0.84(0.82-0.86) | 0.54(0.50-0.58) | 0.98(0.90-1.06) |
| 9 | 0.67(0.64-0.70) | 2.06(1.88-2.27) | 1.84(1.67-2.03) | 0.86(0.84-0.87) | 3.6(3.32-3.90) | 2.50(2.30-2.71) |
| 10 | 0.66(0.63-0.69) | 0.34(0.31-0.37) | 7.80(7.09-8.59) | 0.83(0.82-0.85) | 0.64(0.59-0.69) | 13.93(12.85-15.1) |
| 11 | 0.68(0.65-0.71) | 2.43(2.2-2.67) | 1.34(1.22-1.47) | 0.85(0.83-0.86) | 4.22(3.90-4.58) | 1.40(1.30-1.52) |
| 12 | 0.69(0.66-0.71) | 0.52(0.48-0.58) | 2.56(2.33-2.82) | 0.87(0.85-0.88) | 0.85(0.78-0.92) | 2.62(2.42-2.84) |
| 13 | 0.53(0.5-0.56) | 0.12(0.11-0.14) | 1.24(1.13-1.37) | 0.64(0.61-0.66) | 0.29(0.26-0.31) | 1.18(1.09-1.28) |
| 14 | 0.66(0.63-0.69) | NA | 1.46(1.32-1.60) | 0.86(0.84-0.87) | NA | 1.58(1.46-1.72) |
| 15 | 0.67(0.64-0.70) | 0.17(0.16-0.19) | 1.54(1.40-1.69) | 0.84(0.83-0.86) | 0.26(0.24-0.29) | 1.93(1.78-2.09) |
| 16 | **0.72(0.69-0.75)** | 1.12(1.01-1.23) | 1.93(1.76-2.13) | 0.88(0.86-0.89) | 1.05(0.97-1.14) | 1.98(1.83-2.15) |
| 17 | 0.64(0.61-0.67) | NA | 1.45(1.32-1.60) | 0.79(0.77-0.81) | NA | 2.34(2.16-2.54) |
| 19 | **0.72(0.69-0.74)** | NA | 1.93(1.75-2.12) | 0.85(0.84-0.87) | NA | 1.89(1.74-2.04) |
| 20 | 0.65(0.62-0.68) | 0.15(0.14-0.16) | 1.54(1.40-1.69) | 0.83(0.81-0.85) | 0.24(0.22-0.26) | 1.61(1.49-1.75) |
| 21 | 0.68(0.65-0.71) | 0.30(0.28-0.33) | 1.44(1.31-1.59) | 0.85(0.83-0.86) | 0.76(0.70-0.82) | 1.73(1.59-1.87) |
| 22 | 0.64(0.61-0.68) | 1.48(1.34-1.63) | 1.33(1.21-1.46) | 0.85(0.83-0.87) | 3.08(2.84-3.34) | 1.44(1.33-1.56) |
| 23 | 0.69(0.66-0.71) | 0.33(0.3-0.36) | 2.19(1.99-2.42) | 0.87(0.85-0.88) | 0.62(0.57-0.68) | 2.42(2.24-2.63) |
| 24 | 0.56(0.53-0.59) | 3.63(3.3-3.99) | 1.47(1.33-1.61) | 0.70(0.67-0.72) | 7.70(7.10-8.35) | 1.49(1.38-1.62) |
| 25 | 0.66(0.63-0.69) | 0.34(0.31-0.37) | 0.91(0.83-1.00) | 0.85(0.84-0.87) | 0.68(0.63-0.74) | 0.95(0.87-1.03) |
| 26 | 0.64(0.61-0.67) | 3.32(3.02-3.65) | 1.02(0.93-1.12) | 0.76(0.74-0.78) | 6.37(5.88-6.9) | 1.02(0.94-1.11) |
| 27 | 0.66(0.63-0.69) | 1.24(1.13-1.36) | 1.01(0.92-1.11) | 0.84(0.82-0.86) | 2.09(1.93-2.27) | 1.02(0.94-1.10) |
| 28 | 0.64(0.61-0.67) | NA | 1.36(1.23-1.5) | 0.81(0.8-0.83) | NA | 1.42(1.31-1.54) |
| 29 | 0.61(0.58-0.64) | 0.66(0.6-0.72) | 1.54(1.4-1.69) | 0.75(0.73-0.77) | 1.53(1.41-1.66) | 2.08(1.92-2.25) |
| 30 | 0.64(0.61-0.67) | 0.93(0.84-1.02) | 1.17(1.06-1.28) | 0.83(0.82-0.85) | 1.62(1.49-1.75) | 1.16(1.07-1.25) |
| 31 | 0.65(0.62-0.68) | 1.48(1.35-1.63) | 1.02(0.93-1.13) | 0.83(0.82-0.85) | 2.61(2.41-2.83) | 1.03(0.95-1.12) |
| 32 | 0.66(0.63-0.69) | 0.47(0.43-0.52) | 1.36(1.24-1.5) | 0.84(0.82-0.86) | 0.86(0.79-0.93) | 1.55(1.43-1.68) |
| 33 | 0.68(0.65-0.71) | 0.56(0.51-0.62) | 1.04(0.94-1.14) | 0.81(0.79-0.82) | 1.14(1.05-1.24) | 1.03(0.95-1.11) |
| 34 | 0.67(0.64-0.7) | 1.45(1.32-1.59) | 1.06(0.96-1.17) | 0.83(0.81-0.85) | 1.98(1.83-2.15) | 1.07(0.99-1.16) |
| 35 | 0.67(0.64-0.7) | 1.17(1.06-1.29) | 1.01(0.92-1.11) | 0.84(0.82-0.86) | 2.17(2.00-2.35) | 1.02(0.94-1.10) |
| 36 | 0.68(0.65-0.71) | 1.26(1.14-1.38) | 1.04(0.94-1.14) | 0.85(0.83-0.86) | 1.74(1.60-1.88) | 1.06(0.98-1.15) |
| 37 | 0.68(0.65-0.71) | 2.90(2.63-3.19) | 1.03(0.93-1.13) | 0.85(0.83-0.87) | 3.96(3.66-4.3) | 1.04(0.96-1.13) |
| 38 | 0.65(0.62-0.68) | 0.65(0.59-0.72) | 1.00(0.91-1.1) | 0.77(0.75-0.79) | 1.45(1.34-1.57) | 1.00(0.92-1.08) |
| 39 | 0.70(0.67-0.73) | 1.37(1.24-1.5) | 1.05(0.96-1.16) | 0.86(0.84-0.88) | 1.53(1.41-1.66) | 1.07(0.98-1.16) |
| 40 | 0.64(0.61-0.67) | 1.82(1.66-2.01) | 1.01(0.92-1.11) | 0.83(0.81-0.84) | 2.84(2.62-3.08) | 1.02(0.94-1.10) |
| 41 | 0.71(0.68-0.74) | 0.85(0.78-0.94) | 1.06(0.96-1.17) | 0.87(0.86-0.89) | 1.06(0.98-1.15) | 0.97(0.89-1.05) |
| 43 | **0.72(0.70-0.75)** | 0.72(0.65-0.79) | 1.30(1.18-1.43) | **0.89(0.88-0.90)** | 0.66(0.61-0.71) | 1.32(1.22-1.43) |
| 44 | 0.67(0.64-0.7) | 0.30(0.27-0.33) | 1.75(1.59-1.93) | 0.86(0.85-0.88) | 0.83(0.77-0.90) | 2.38(2.19-2.58) |
| 45 | 0.68(0.65-0.71) | 0.89(0.81-0.98) | 1.13(1.03-1.24) | 0.84(0.82-0.86) | 1.06(0.97-1.15) | 1.16(1.07-1.25) |
| 47 | 0.68(0.66-0.71) | 0.53(0.48-0.58) | 1.05(0.95-1.16) | 0.85(0.83-0.87) | 0.64(0.59-0.70) | 1.09(1.00-1.18) |
| 48 | 0.67(0.65-0.70) | 0.99(0.9-1.09) | 1.30(1.18-1.43) | 0.84(0.82-0.86) | 1.44(1.32-1.56) | 1.54(1.42-1.67) |
| 49 | 0.68(0.65-0.70) | 2.18(1.98-2.4) | 1.06(0.97-1.17) | 0.83(0.81-0.84) | 3.75(3.46-4.06) | 1.10(1.01-1.19) |

CI: Confidence interval. NA: not applicable.

**Table S11** The validation results of the included BASIC models in intermediate hyperglycemia participants of the ACE, Luzhou, and TCLSIH cohorts when using complete cases for analysis.

| **Study**  **No.** | **ACE cohort** | | | **Luzhou cohort** | | | **TCLSIH cohort** | | |
| --- | --- | --- | --- | --- | --- | --- | --- | --- | --- |
|  | **C-statistic**  **(95% CI)** | **Original**  **P/0 (95% CI)** | **Recalibrated P/0 (95% CI)** | **C-statistic**  **(95% CI)** | **Original**  **P/0 (95% CI)** | **Recalibrated P/0 (95%CI)** | **C-statistic**  **(95% CI)** | **Original**  **P/0 (95%CI)** | **Recalibrated P/0 (95% CI)** |
| 1 | 0.58(0.55-0.6) | NA | 1.34(1.23-1.46) | 0.57(0.52-0.61) | NA | 1.33(1.17-1.52) | 0.63(0.6-0.67) | NA | 1.43(1.29-1.59) |
| 3 | 0.59(0.56-0.61) | 1.07(0.98-1.16) | 1.15(1.06-1.25) | 0.54(0.49-0.58) | 0.34(0.29-0.39) | 1.15(1.01-1.32) | 0.59(0.55-0.62) | 0.46(0.41-0.51) | 1.14(1.03-1.27) |
| 4 | 0.56(0.54-0.59) | NA | 1.25(1.15-1.36) | 0.58(0.54-0.63) | NA | 1.31(1.15-1.5) | 0.62(0.58-0.65) | NA | 1.33(1.21-1.48) |
| 5 | 0.56(0.54-0.59) | 0.39(0.36-0.42) | 1(0.92-1.09) | 0.57(0.52-0.61) | 0.2(0.17-0.22) | 1(0.88-1.15) | 0.63(0.59-0.62) | 0.22(0.2-0.25) | 1(0.9-1.11) |
| 6 | 0.52(0.5-0.55) | NA | 1.53(1.41-1.67) | 0.58(0.53-0.63) | NA | 1.32(1.15-1.51) | 0.55(0.51-0.59) | NA | 1.29(1.17-1.44) |
| 7 | 0.59(0.57-0.62) | 0.38(0.35-0.41) | 1.19(1.1-1.3) | 0.56(0.51-0.61) | 0.15(0.13-0.17) | 1.16(1.02-1.33) | 0.62(0.58-0.65) | 0.18(0.17-0.2) | 1.21(1.09-1.35) |
| 8 | 0.59(0.56-0.62) | 0.51(0.47-0.56) | 0.99(0.91-1.08) | 0.55(0.50-0.60) | 0.11(0.09-0.12) | 0.93(0.81-1.06) | 0.6(0.56-0.64) | 0.19(0.17-0.21) | 0.95(0.85-1.05) |
| 9 | 0.57(0.54-0.59) | NA | 1.16(1.07-1.27) | 0.59(0.54-0.64) | NA | 1.17(1.02-1.34) | 0.63(0.59-0.66) | NA | 1.21(1.09-1.34) |
| 12 | 0.58(0.56-0.61) | 1.05(0.97-1.15) | 1.28(1.18-1.39) | 0.58(0.53-0.62) | 0.31(0.27-0.35) | 1.34(1.18-1.54) | 0.59(0.55-0.63) | 0.42(0.38-0.47) | 1.40(1.27-1.56) |
| 13 | 0.58(0.55-0.61) | NA | 1.1(1.02-1.2) | 0.54(0.50-0.59) | NA | 1.12(0.98-1.29) | 0.61(0.57-0.64) | NA | 1.13(1.02-1.25) |
| 16 | 0.52(0.5-0.55) | 1.36(1.25-1.48) | 1.18(1.09-1.29) | 0.58(0.53-0.62) | 0.28(0.25-0.32) | 1.18(1.03-1.35) | 0.62(0.58-0.65) | 0.33(0.29-0.36) | 1.17(1.05-1.3) |
| 18 | 0.55(0.53-0.58) | NA | 1.27(1.16-1.38) | 0.57(0.52-0.61) | NA | 1.41(1.24-1.61) | 0.63(0.6-0.67) | NA | 1.45(1.31-1.61) |
| 23 | 0.59(0.57-0.62) | 0.92(0.84-1) | 1.24(1.14-1.35) | 0.57(0.53-0.62) | 0.25(0.22-0.28) | 1.24(1.09-1.42) | 0.58(0.55-0.62) | 0.37(0.33-0.41) | 1.3(1.17-1.44) |
| 30 | 0.54(0.51-0.57) | 2.62(2.41-2.85) | 1.04(0.96-1.13) | 0.56(0.51-0.61) | 0.46(0.4-0.53) | 1.11(0.98-1.27) | 0.63(0.59-0.67) | 0.45(0.4-0.5) | 1.1(0.99-1.22) |
| 33 | 0.56(0.53-0.59) | 0.79(0.72-0.86) | 1(0.92-1.09) | 0.55(0.51-0.60) | 0.32(0.28-0.37) | 1(0.88-1.15) | 0.56(0.52-0.59) | 0.37(0.34-0.41) | 1.00(0.91-1.12) |
| 41 | NA^*^ | NA^*^ | NA^*^ | 0.50(0.46-0.55) | 1.12(0.98-1.28) | NA | 0.51(0.47-0.55) | 1.02(0.92-1.14) | 1.00(0.9-1.11) |
| 42 | 0.56(0.53-0.59) | 1.38(1.27-1.5) | 1.02(0.94-1.11) | 0.58(0.53-0.63) | 0.31(0.27-0.36) | 0.96(0.84-1.09) | 0.63(0.6-0.67) | 0.4(0.36-0.44) | 0.98(0.88-1.09) |
| 44 | 0.54(0.52-0.57) | 1.75(1.61-1.9) | 1.64(1.51-1.79) | 0.58(0.53-0.63) | 0.13(0.11-0.15) | 1.23(1.08-1.42) | 0.63(0.59-0.66) | 0.3(0.28-0.34) | 1.29(1.17-1.44) |
| 46 | 0.56(0.53-0.59) | 0.11(0.1-0.12) | 0.93(0.85-1.01) | 0.56(0.52-0.61) | 0.03(0.03-0.03) | 0.92(0.81-1.06) | 0.58(0.54-0.61) | 0.04(0.04-0.05) | 0.87(0.79-0.97) |
| 47 | 0.53(0.5-0.56) | 1.07(0.98-1.17) | 1.03(0.95-1.13) | 0.60(0.55-0.64) | 0.26(0.23-0.3) | 0.98(0.86-1.12) | 0.61(0.58-0.65) | 0.27(0.24-0.3) | 0.98(0.89-1.09) |
| 48 | 0.58(0.56-0.61) | 0.58(0.53-0.63) | 0.99(0.91-1.08) | 0.57(0.52-0.61) | 0.13(0.11-0.14) | 0.95(0.83-1.09) | 0.59(0.56-0.63) | 0.18(0.16-0.2) | 0.95(0.86-1.06) |

^*:^ The validation in ACE cohort was not conducted for this study because it was developed based on the ACE cohort. CI: Confidence interval. NA: not applicable.

**Table S12** The validation results of the included EXTENDED models in intermediate hyperglycemia participants of the ACE, Luzhou, and TCLSIH cohorts when using complete cases for analysis.

| **Study**  **No.** | **ACE cohort** | | | **Luzhou cohort** | | | **TCLSIH cohort** | | | |
| --- | --- | --- | --- | --- | --- | --- | --- | --- | --- | --- |
|  | **C-statistic**  **(95% CI)** | **Original**  **P/0 (95% CI)** | **Recalibrated P/0 (95% CI)** | **C-statistic**  **(95% CI)** | **Original**  **P/0 (95% CI)** | **Recalibrated P/0 (95% CI)** | **C-statistic**  **(95% CI)** | **Original**  **P/0 (95% CI)** | **Recalibrated P/0 (95% CI)** |  |
| 1 | 0.59(0.56-0.62) | NA | 1.35(1.24-1.47) | 0.55(0.5-0.59) | NA | 1.46(1.28-1.67) | 0.61(0.58-0.65) | NA | 1.65(1.49-1.83) |  |
| 2 | 0.68(0.66-0.71) | 1.15(1.05-1.25) | 1.29(1.18-1.4) | 0.61(0.56-0.66) | 0.54(0.48-0.62) | 1.23(1.08-1.41) | 0.68(0.65-0.72) | 0.56(0.5-0.62) | 1.32(1.19-1.46) |  |
| 3 | 0.68(0.65-0.71) | NA | 1.42(1.3-1.54) | 0.59(0.54-0.64) | NA | 1.38(1.21-1.58) | 0.70(0.67-0.73) | NA | 1.41(1.27-1.57) |  |
| 4 | 0.69(0.66-0.71) | 1.11(1.02-1.21) | 2.34(2.16-2.55) | 0.62(0.58-0.67) | 0.30(0.26-0.35) | 1.84(1.61-2.1) | 0.73(0.7-0.76) | 0.44(0.4-0.49) | 2.28(2.06-2.54) |  |
| 5 | 0.67(0.65-0.70) | 0.67(0.61-0.72) | 1(0.92-1.09) | 0.61(0.56-0.66) | 0.36(0.31-0.41) | 1(0.88-1.15) | 0.72(0.68-0.75) | 0.38(0.34-0.42) | 1(0.9-1.11) |  |
| 6 | 0.63(0.61-0.66) | NA | 1.24(1.14-1.35) | 0.61(0.56-0.65) | NA | 1.23(1.08-1.41) | 0.68(0.65-0.72) | NA | 1.17(1.05-1.3) |  |
| 7 | 0.68(0.65-0.71) | 0.4(0.37-0.44) | 1.49(1.37-1.63) | 0.59(0.54-0.64) | 0.11(0.1-0.13) | 1.36(1.19-1.56) | 0.71(0.68-0.75) | 0.16(0.14-0.17) | 1.49(1.35-1.66) |  |
| 8 | 0.69(0.66-0.71) | 0.74(0.68-0.81) | 0.99(0.91-1.08) | 0.6(0.55-0.64) | 0.31(0.27-0.35) | 0.92(0.81-1.06) | 0.73(0.7-0.76) | 0.34(0.31-0.38) | 0.9(0.82-1) |  |
| 9 | 0.68(0.66-0.71) | 2.75(2.53-2.99) | 1.76(1.62-1.92) | 0.62(0.57-0.66) | 1.14(1-1.31) | 1.56(1.36-1.78) | 0.73(0.7-0.76) | 1.11(1-1.23) | 1.75(1.58-1.94) |  |
| 10 | 0.67(0.64-0.69) | 0.89(0.82-0.97) | 5.69(5.24-6.2) | 0.6(0.56-0.65) | 0.27(0.24-0.31) | 4.48(3.92-5.13) | 0.71(0.68-0.74) | 0.32(0.29-0.36) | 4.08(3.68-4.53) |  |
| 11 | 0.7(0.67-0.72) | 3.35(3.08-3.65) | 1.34(1.24-1.46) | 0.64(0.59-0.68) | 1.5(1.32-1.72) | 1.29(1.13-1.48) | 0.72(0.69-0.75) | 1.29(1.17-1.44) | 1.39(1.26-1.55) |  |
| 12 | 0.71(0.68-0.73) | 1.37(1.26-1.5) | 2.4(2.21-2.61) | 0.64(0.6-0.69) | 0.52(0.46-0.6) | 2.16(1.9-2.48) | 0.74(0.71-0.77) | 0.66(0.59-0.73) | 2.1(1.89-2.33) |  |
| 13 | 0.6(0.57-0.63) | 0.17(0.15-0.18) | 1.3(1.2-1.42) | 0.52(0.47-0.56) | 0.07(0.06-0.08) | 1.22(1.07-1.4) | 0.56(0.53-0.6) | 0.08(0.07-0.09) | 1.19(1.08-1.32) |  |
| 14 | 0.68(0.65-0.71) | NA | 1.46(1.34-1.59) | 0.60(0.55-0.65) | NA | 1.42(1.25-1.63) | 0.72(0.69-0.75) | NA | 1.48(1.34-1.64) |  |
| 15 | 0.67(0.64-0.69) | 0.27(0.25-0.3) | 1.38(1.27-1.5) | 0.61(0.56-0.66) | 0.13(0.12-0.15) | 1.37(1.20-1.57) | 0.7(0.67-0.73) | 0.13(0.12-0.15) | 1.44(1.3-1.6) |  |
| 16 | **0.73(0.7-0.75)** | 3.53(3.24-3.84) | 1.82(1.67-1.98) | 0.67(0.63-0.72) | 1.21(1.06-1.38) | 1.56(1.36-1.78) | 0.75(0.72-0.78) | 0.92(0.83-1.03) | 1.55(1.4-1.72) |  |
| 17 | 0.66(0.63-0.68) | NA | 1.51(1.39-1.65) | 0.61(0.56-0.65) | NA | 1.44(1.27-1.66) | 0.69(0.65-0.72) | NA | 1.71(1.55-1.9) |  |
| 19 | 0.69(0.67-0.72) | 0.81(0.74-0.88) | 1.52(1.39-1.65) | 0.64(0.59-0.68) | 0.46(0.4-0.53) | 1.54(1.35-1.77) | 0.68(0.64-0.71) | 0.4(0.37-0.45) | 1.5(1.35-1.66) |  |
| 20 | 0.67(0.64-0.7) | 0.29(0.26-0.31) | 1.55(1.43-1.69) | 0.6(0.55-0.65) | 0.14(0.12-0.16) | 1.5(1.32-1.72) | 0.69(0.66-0.73) | 0.17(0.16-0.19) | 1.57(1.42-1.75) |  |
| 21 | 0.63(0.6-0.66) | 1.31(1.21-1.43) | 1.73(1.59-1.88) | 0.63(0.58-0.68) | 0.23(0.2-0.26) | 1.34(1.18-1.54) | 0.71(0.68-0.74) | 0.35(0.32-0.39) | 1.4(1.27-1.56) |  |
| 22 | 0.68(0.65-0.71) | 2.22(2.04-2.42) | 1.41(1.3-1.53) | 0.6(0.55-0.65) | 0.91(0.8-1.04) | 1.32(1.16-1.51) | 0.71(0.68-0.75) | 0.95(0.86-1.06) | 1.4(1.27-1.56) |  |
| 23 | 0.71(0.69-0.74) | 0.73(0.67-0.8) | 2.08(1.91-2.26) | 0.64(0.6-0.69) | 0.33(0.29-0.37) | 1.91(1.68-2.19) | 0.74(0.71-0.77) | 0.48(0.43-0.53) | 1.96(1.77-2.18) |  |
| 24 | 0.56(0.53-0.58) | 5.38(4.95-5.85) | 1.34(1.23-1.46) | 0.54(0.49-0.59) | 2(1.75-2.29) | 1.39(1.22-1.59) | 0.61(0.58-0.65) | 1.94(1.76-2.16) | 1.35(1.22-1.5) |  |
| 25 | 0.68(0.66-0.71) | 0.71(0.65-0.77) | 0.92(0.84-1) | 0.61(0.56-0.66) | 0.31(0.28-0.36) | 0.87(0.76-1) | 0.72(0.69-0.75) | 0.48(0.43-0.53) | 0.88(0.79-0.98) |  |
| 26 | 0.6(0.57-0.62) | 4.67(4.3-5.09) | 1.01(0.93-1.1) | 0.61(0.56-0.65) | 2.19(1.92-2.51) | 1.02(0.89-1.16) | 0.64(0.6-0.67) | 2.24(2.02-2.49) | 1.01(0.92-1.13) |  |
| 27 | 0.66(0.64-0.69) | 1.82(1.67-1.98) | 1.01(0.93-1.1) | 0.62(0.58-0.67) | 0.88(0.77-1.01) | 1.01(0.88-1.16) | 0.70(0.67-0.73) | 0.9(0.81-1) | 1.01(0.91-1.12) |  |
| 28 | 0.67(0.64-0.69) | NA | 1.34(1.23-1.46) | 0.59(0.54-0.64) | NA | 1.33(1.17-1.53) | 0.68(0.65-0.72) | NA | 1.34(1.21-1.49) |  |
| 29 | 0.54(0.52-0.57) | 2.49(2.29-2.72) | 1.3(1.19-1.41) | 0.58(0.54-0.63) | 0.44(0.38-0.5) | 1.35(1.18-1.55) | 0.62(0.58-0.66) | 0.57(0.51-0.63) | 1.4(1.26-1.55) |  |
| 30 | 0.64(0.61-0.66) | 2.49(2.29-2.71) | 1.15(1.06-1.25) | 0.58(0.54-0.63) | 0.67(0.59-0.77) | 1.14(1-1.31) | 0.73(0.69-0.76) | 0.65(0.59-0.72) | 1.13(1.02-1.26) |  |
| 31 | 0.65(0.63-0.68) | 2.54(2.33-2.76) | 1.02(0.94-1.11) | 0.62(0.57-0.67) | 1.09(0.95-1.25) | 1.02(0.89-1.17) | 0.70(0.66-0.73) | 1.24(1.12-1.38) | 1.02(0.92-1.14) |  |
| 32 | 0.68(0.66-0.71) | 0.78(0.72-0.85) | 1.28(1.18-1.39) | 0.61(0.57-0.66) | 0.33(0.29-0.38) | 1.24(1.09-1.42) | 0.70(0.67-0.73) | 0.35(0.31-0.38) | 1.32(1.19-1.47) |  |
| 33 | 0.7(0.67-0.72) | 0.67(0.62-0.73) | 1.05(0.97-1.15) | 0.64(0.6-0.69) | 0.31(0.27-0.35) | 1.04(0.91-1.19) | 0.70(0.67-0.74) | 0.28(0.26-0.32) | 1.04(0.94-1.16) |  |
| 34 | 0.66(0.63-0.68) | 2.55(2.34-2.77) | 1.05(0.96-1.14) | 0.61(0.57-0.66) | 0.97(0.85-1.12) | 1.04(0.91-1.19) | 0.71(0.68-0.75) | 0.79(0.71-0.88) | 1.05(0.95-1.16) |  |
| 35 | 0.67(0.64-0.7) | 1.69(1.55-1.84) | 1.01(0.93-1.1) | 0.62(0.57-0.67) | 0.82(0.72-0.94) | 1.01(0.88-1.16) | 0.71(0.68-0.75) | 0.81(0.73-0.9) | 1.01(0.92-1.13) |  |
| 36 | 0.71(0.68-0.73) | 1.99(1.83-2.17) | 1.04(0.96-1.13) | 0.65(0.6-0.69) | 0.9(0.79-1.03) | 1.03(0.9-1.18) | 0.74(0.71-0.77) | 0.78(0.7-0.86) | 1.05(0.94-1.16) |  |
| 37 | 0.69(0.67-0.72) | 4.76(4.38-5.18) | 1.03(0.95-1.12) | 0.66(0.61-0.71) | 1.77(1.56-2.03) | 1.02(0.89-1.17) | 0.75(0.72-0.78) | 1.38(1.24-1.53) | 1.03(0.93-1.14) |  |
| 38 | 0.62(0.59-0.64) | 1.42(1.31-1.55) | 1(0.92-1.09) | 0.58(0.53-0.63) | 0.42(0.37-0.49) | 1.00(0.88-1.15) | 0.65(0.61-0.68) | 0.51(0.46-0.56) | 1.00(0.90-1.11) |  |
| 39 | 0.72(0.7-0.75) | 2.25(2.07-2.45) | 1.05(0.97-1.14) | 0.66(0.62-0.71) | 1.13(0.99-1.29) | 1.03(0.9-1.18) | 0.76(0.73-0.79) | 0.79(0.72-0.88) | 1.05(0.95-1.17) |  |
| 40 | 0.66(0.63-0.69) | 2.92(2.68-3.18) | 1.01(0.93-1.1) | 0.61(0.56-0.66) | 1.36(1.20-1.56) | 1.01(0.89-1.16) | 0.70(0.66-0.73) | 1.33(1.2-1.47) | 1.02(0.92-1.13) |  |
| 41 | NA^*^ | NA^*^ | NA^*^ | 0.64(0.6-0.69) | 0.85(0.74-0.97) | 1.05(0.92-1.2) | 0.74(0.71-0.78) | 0.71(0.64-0.79) | 0.94(0.85-1.04) |  |
| 43 | **0.73(0.71-0.76)** | 1.71(1.58-1.87) | 1.32(1.22-1.44) | **0.67(0.63-0.72)** | 0.69(0.60-0.79) | 1.20(1.05-1.37) | **0.78(0.75-0.81)** | 0.48(0.43-0.53) | 1.20(1.08-1.33) |  |
| 44 | 0.67(0.64-0.69) | 1.78(1.64-1.94) | 2.14(1.97-2.33) | 0.63(0.59-0.68) | 0.25(0.22-0.29) | 1.56(1.37-1.79) | 0.73(0.7-0.76) | 0.44(0.4-0.49) | 1.77(1.6-1.96) |  |
| 45 | 0.66(0.63-0.69) | 1.76(1.62-1.92) | 1.25(1.15-1.36) | 0.62(0.57-0.66) | 0.67(0.59-0.77) | 1.09(0.95-1.25) | 0.71(0.68-0.75) | 0.53(0.48-0.59) | 1.04(0.94-1.16) |  |
| 47 | 0.68(0.65-0.7) | 1.16(1.06-1.26) | 1.17(1.08-1.27) | 0.62(0.57-0.67) | 0.43(0.37-0.49) | 1.02(0.9-1.17) | 0.72(0.69-0.76) | 0.38(0.34-0.42) | 1.00(0.9-1.11) |  |
| 48 | 0.68(0.66-0.71) | 1.92(1.77-2.09) | 1.51(1.39-1.64) | 0.61(0.56-0.66) | 0.80(0.70-0.92) | 1.25(1.1-1.44) | 0.72(0.68-0.75) | 0.81(0.73-0.9) | 1.36(1.22-1.51) |  |
| 49 | 0.66(0.64-0.69) | 4.86(4.47-5.29) | 1.06(0.98-1.16) | 0.63(0.58-0.68) | 1.39(1.22-1.59) | 1.04(0.92-1.2) | 0.69(0.66-0.73) | 1.33(1.2-1.48) | 1.05(0.95-1.17) |  |

^*:^ The validation in ACE cohort was not conducted for this study because it was developed based on the ACE cohort. CI: Confidence interval. NA: not applicable.
